# Of cattle and feasts: Multi-isotope investigation of animal husbandry and communal feasting at Neolithic Makriyalos, northern Greece

**DOI:** 10.1371/journal.pone.0194474

**Published:** 2018-06-07

**Authors:** Petra Vaiglova, Paul Halstead, Maria Pappa, Sevi Triantaphyllou, Soultana M. Valamoti, Jane Evans, Rebecca Fraser, Panagiotis Karkanas, Andrea Kay, Julia Lee-Thorp, Amy Bogaard

**Affiliations:** 1 Research Laboratory for Archaeology and the History of Art, University of Oxford, Oxford, United Kingdom; 2 Wiener Laboratory for Archaeological Science, American School of Classical Studies, Athens, Greece; 3 Department of Archaeology, University of Sheffield, Sheffield, United Kingdom; 4 Ephorea of Pieria, Hellenic Ministry of Education and Religious Affairs, Culture and Sports, Thessaloniki, Greece; 5 School of History and Archaeology, Aristotle University of Thessaloniki, Thessaloniki, Greece; 6 Natural Environment Research Council Isotope Geoscience Laboratory, British Geological Survey, Keyworth, United Kingdom; 7 Institute of Archaeology, University of Oxford, Oxford, United Kingdom; 8 Institute of Earth Surface Dynamics, University of Lausanne, Lausanne, Switzerland; University of Otago, NEW ZEALAND

## Abstract

The aim of this study is to investigate livestock husbandry and its relationship to the mobilization of domestic animals for slaughter at large communal feasting events, in Late Neolithic Makriyalos, northern Greece. A multi-isotope approach is built that integrates analysis of:

1. δ^13^C and δ^15^N values of human and animal bone collagen for understanding long-term dietary behavior,
2. Incremental δ^13^C and δ^18^O values of domestic animal tooth enamel carbonate for assessing seasonal patterns in grazing habits and mobility, and
3. ^87^Sr/^86^Sr ratios of cattle tooth enamel for examining the possibility that some of the animals consumed at the site were born outside the local environment.

The findings indicate that cattle had isotopically more variable diets than sheep, which may reflect grazing over a wider catchment area in the local landscape. Cattle products did not make a significant contribution to the long-term dietary protein intake of the humans, which may indicate that they were primarily consumed during episodic feasting events. There is no indication that pasturing of livestock was pre-determined by their eventual context of slaughter (i.e. large-scale feasting vs. more routine consumption events). Two non-local cattle identified among those deposited in a feasting context may have been brought to the site as contributions to these feasts. The evidence presented provides a more detailed insight into local land use and into the role of livestock and feasting in forging social relationships within the regional human population.

## Introduction

**Fig 1:**
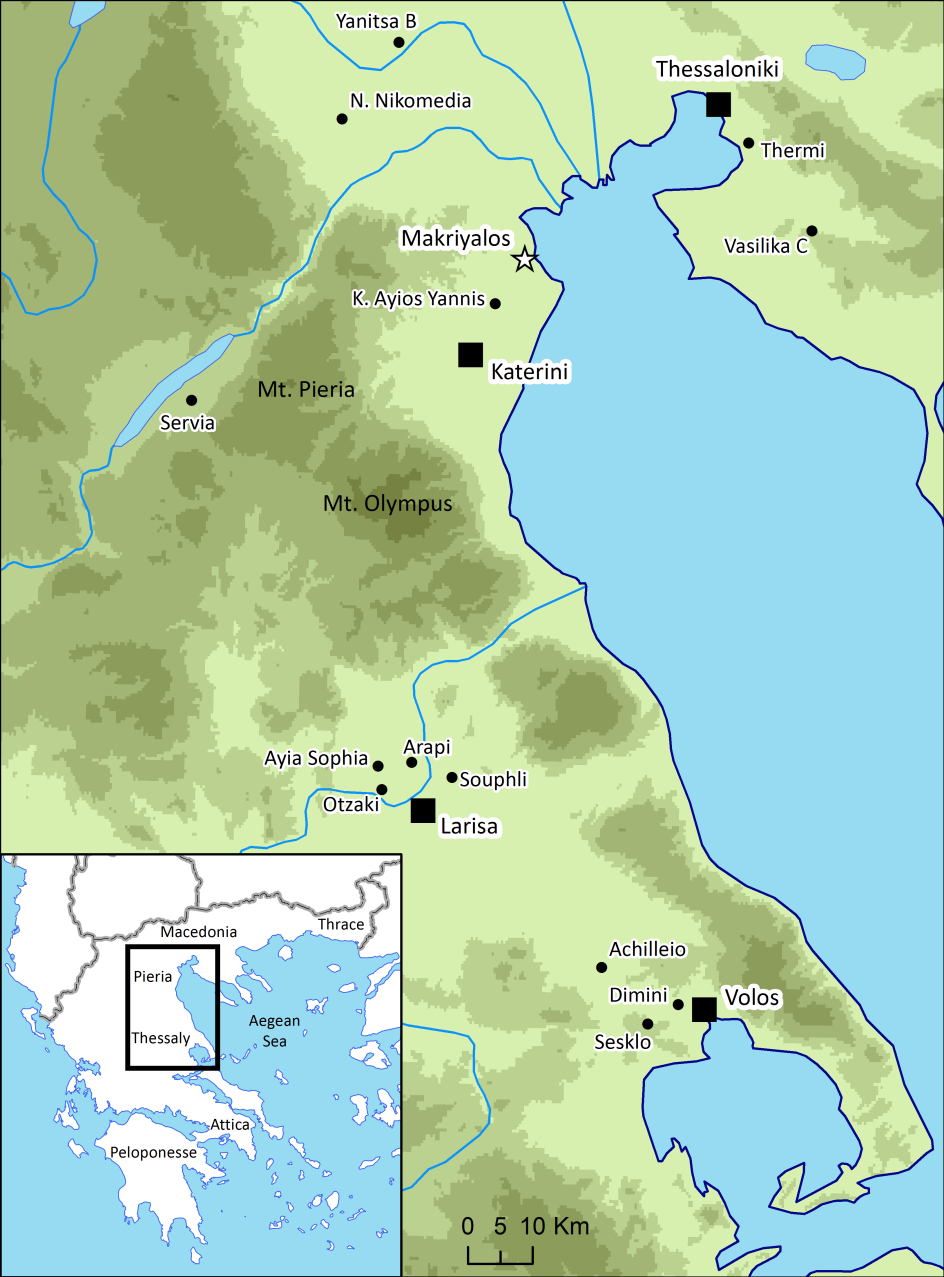
Location of the archaeological site. Makriyalos is shown in relation to modern cities and other Neolithic sites from the region. Map prepared in ArcGIS 10.2 using Digital Elevation Model from Yamazaki et al. [6].

The scale of livestock husbandry and the contribution of domestic animal carcasses to communal feasting activities are two themes that play prominent roles in archaeological discourse on the development of early farming societies in Western Eurasia and beyond [1–4]. The archaeological site of Makriyalos I (MKI), located in the coastal region of Greek Macedonia (Fig 1) and dating to the earlier Late Neolithic (5500–5000/4900 cal BC [5]), provides an exceptional opportunity to assess the interplay of these two elements. One rapidly formed refuse pit excavated at the site (Pit 212) contained unusually large quantities of consumption debris. These included butchered bones and teeth of several hundred mainly domestic animals (at least 600 individuals from the basal layer of just the excavated half of the pit, representing tens of tons of meat [4]) and a comparable wealth of ceramic (mainly table and cooking ware) vessels, implying that feasting activities took place here on an unparalleled scale. It is the aim of this study to shed light on the lives of animals that were consumed during these and other events in order to explore how animal management was adapted to the local environment and how it enabled the organization of large-scale feasting in this early farming community.

To this end, a multi-isotopic study was designed and carried out in stages, with the findings from each stage informing sampling for the next. The results are herein interpreted in light of previous findings from faunal, lithic and organic residue analyses. First, long-term patterns in the animals’ grazing behavior–specifically in terms of the contribution of C_3_ and C_4_ plants to their diets–are assessed using measurements of bone collagen δ^13^C and δ^15^N values. Secondly, their seasonal dietary and mobility patterns are investigated through the analysis of incremental tooth enamel carbonate δ^13^C and δ^18^O values. Lastly, given the apparently large-scale animal slaughter at this site [4], the possibility that non-local stock were among those consumed is tested through an investigation of the animals’ geographical origin using tooth enamel ^87^Sr/^86^Sr ratios. By integrating these datasets, we build a contextualized framework for ascertaining how feasting articulated with the management system employed by farmers at Makriyalos. We thus develop a case study that sheds light on broader questions of how food production provisioned not only the modular households that were the building blocks of Neolithic society, but also a wider regional community [5,7,8].

### The site of Makriyalos

**Fig 2:**
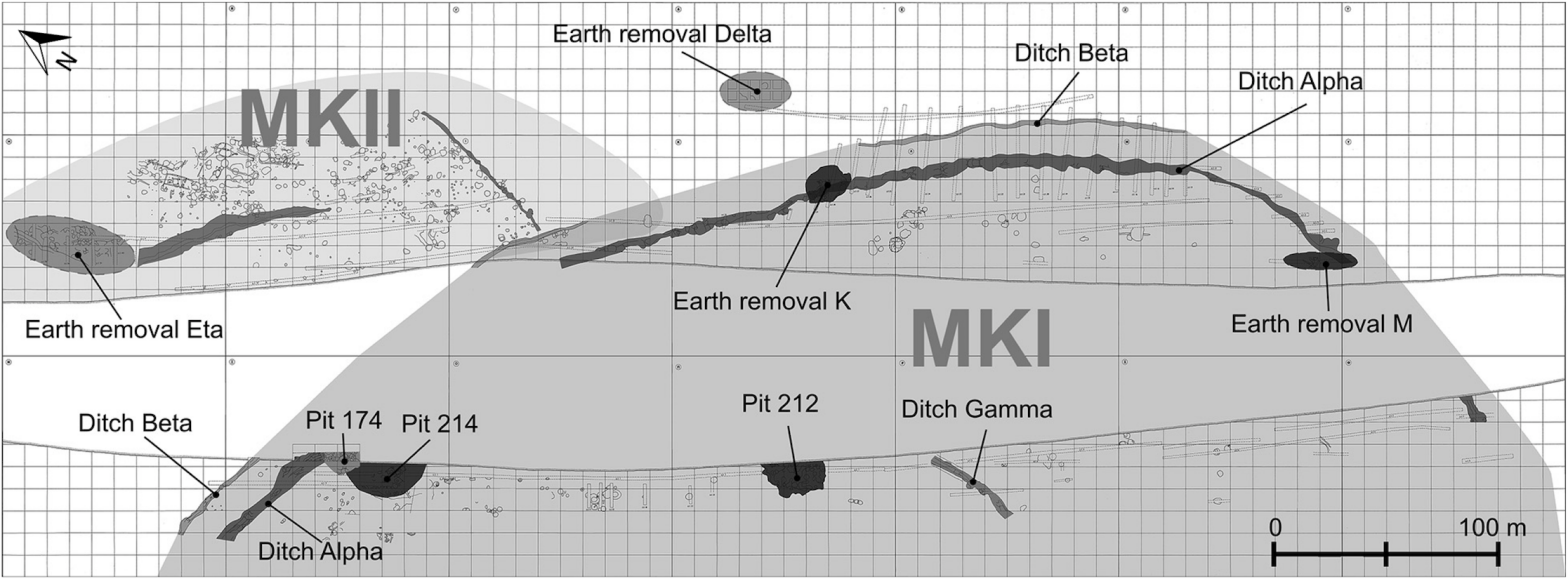
Topographical plan of the two settlements at Makriyalos. MKI: Makriyalos I (early Late Neolithic), MKII: Makriyalos II (late Late Neolithic). Reprinted from Pappa et al. [5] under a CC BY license, with permission from Peeters Publishers, original copyright 2013.

Makriyalos (40 25° 6.32” N, 22° 35’ 30.10” E) is located on the coastal fringe of northern Greece between the Thermaic Gulf (Aegean Sea) 2 km to the east and the Pieria mountains 15 km to the southwest. Rescue excavations and survey carried out in the 1990s revealed that the site consists of two spatially separate settlements occupied in two successive phases (see Fig 2) [9,10]. Makriyalos I dates to the early Late Neolithic (5500–5000/4900 cal BC) and covers an area of 28 ha and Makriyalos II dates to the late Late Neolithic (5000/4900–4500 cal BC) and extends over 11 ha [5]. MKI exemplifies a ‘flat extended’ type of settlement, in which chronological sequences of deposits do not accumulate vertically (as they do at ‘tell’ sites) but are found dispersed horizontally over a wider area [11].

During the Makriyalos I phase, habitation was loosely organized into clusters of semi-subterranean pit-dwellings enclosed by a pair of concentric ditches [9,10]. A large proportion of the material excavated from this period derives from Pit 212, which saw the rapid deposition of large quantities of faunal and artifactual material. This deposit is argued to represent the discard of a series of large-scale feasting activities, which may have taken place over several weeks or months and may have been attended by participants from the wider geographical region [4]. Both the stylistic variability of the ceramic assemblage inside this pit and the large number of ‘individualized’ cups are compatible with the vessels (and participants) being “drawn from a wider region than is represented in other Makriyalos I deposits” (p.38) [4]. The quantity of meat represented by the animal remains in Pit 212 suggests that it was consumed by at least several dozen to a few hundred people (depending on how rapidly these remains were discarded), while traces of butchery and bone breakage suggest that carcass processing and consumption took place in the vicinity of this pit [4]. This archaeological deposit provides an unusual opportunity to explore how animal husbandry practices underpinned Late Neolithic feasting on such a large scale.

Material from Makriyalos I has been studied by a range of specialists, whose work enabled further inquiry into the scale of animal management and its role in the Neolithic economy. Macroscopic faunal analysis used mortality profiles of the major animal domesticates (cattle, sheep, goats, and pigs) to infer that their management was meat–rather than milk–oriented [5], but the seasonal dietary habits of the animals remain to be determined. Bone collagen stable isotopic values of humans from MKI and of domestic pigs and wild mammalian fauna from the later Makriyalos II (wild mammals were rare in the MKI assemblage) showed that marine foods played a minor role in long-term human diets [12], but comparative values of contemporary domestic animals and plants are needed for a fuller and more reliable picture of human dietary habits. Assessment of sheep and goat dental microwear patterns indicated differences in diets between ovicaprids discarded in feasting and non-feasting contexts [13], but the possibility of differential treatment of cattle consumed on small- and large-scale occasions remains to be investigated. Analysis of chipped and ground stone tools suggested that some raw materials may have been brought to the site from non-local sources [5,14], but the hypothesis that other commodities–such as domestic animals–may also have been obtained from a distance [15] remains to be tested. It is the aim of this study to address these research gaps through an investigation of several aspects of the lives of sheep and cattle slaughtered at Makriyalos.

Halstead [7] has suggested that the 28 ha enclosure of Makriyalos I included cultivation plots as well as dwellings, while the abrasive dental microwear of sheep and goats from this settlement may be attributable to grazing of land disturbed by tillage [13]. The enclosure could have sustained (for example) a few dozen sheep all year round or a few hundred sheep for a few months, while the corresponding numbers of cattle would have been far smaller. Assessment of how localized the grazing patterns of the domestic herbivores were may thus shed light on the nature of local and regional land use and the scale of animal husbandry, as well as on the mobilization of animals for commensality.

### The environmental setting

The coastal environment of northern Greece has been in a continuous state of change since the late Pleistocene [16–18]. High-resolution modeling suggests that between 10,000 and 6,000 BP, the mean level of the Thermaic Gulf was 3–46 m lower than today [19] and that, during the Late Neolithic, the shoreline near to Makriyalos was located a few hundred meters east of its present position [16].

**Fig 3:**
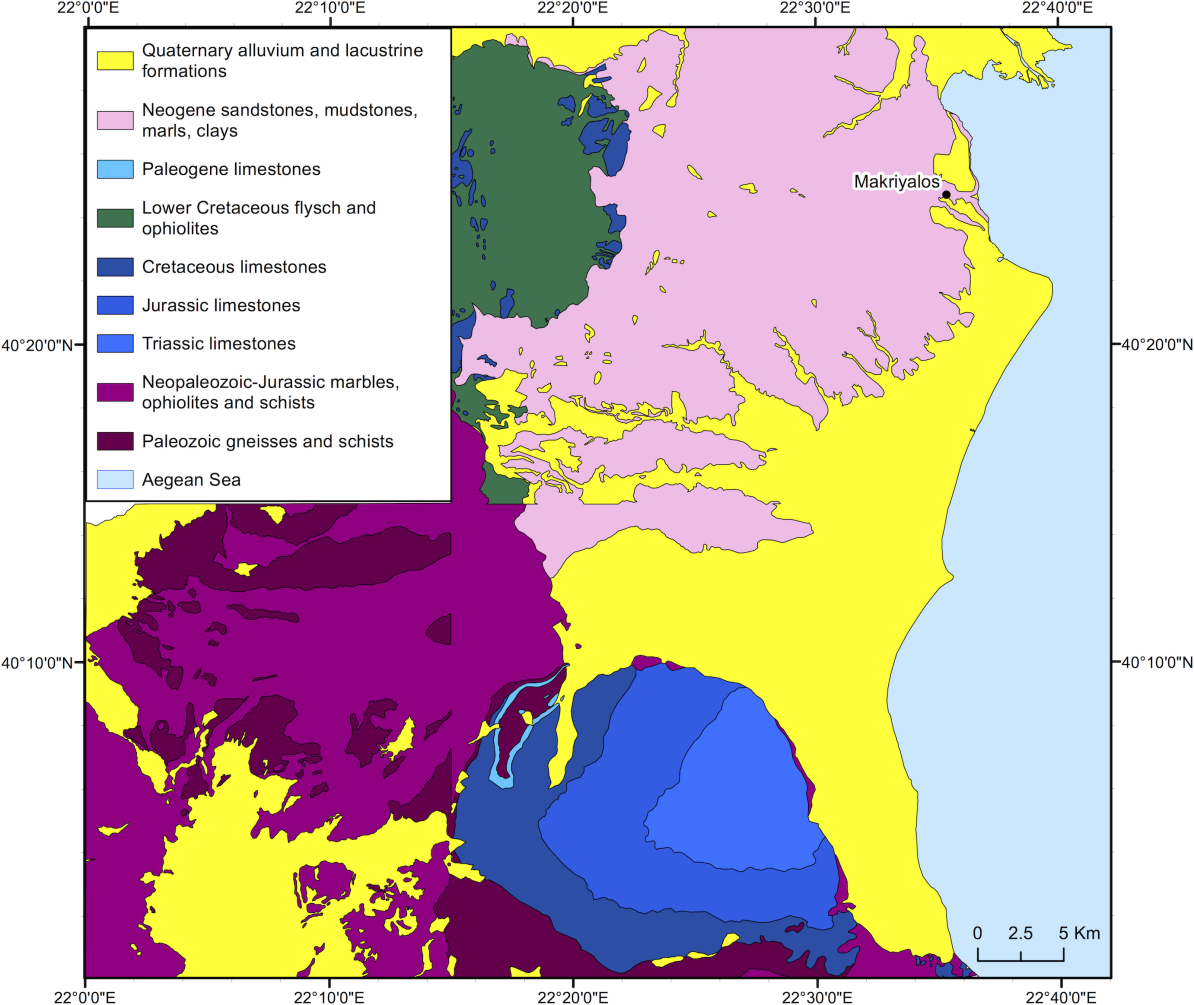
Geological map of Pieria, northern Greece. Prepared using material published by the Greek Institute of Geology and Mineral Exploration [23–26]. The published information was digitized using ArcGIS 10.2 and geo-referenced to the Greek grid.

Makriyalos is situated close to the interface between the Pierian hills, locally formed of Neogene mudstones (see Fig 3), and the modern shoreline. These hills rise to an altitude of 400 masl and form the piedmont of the Pieria Mountains to the west and the Olympus range to the south. Two ravines pass by the site, one to the northeast and one to the southwest [9]. Coastal brackish marshes, where the more saline-adapted C_4_ plants are available and perhaps all year round (cf [20]) are located c.7–8 km to the north and south of the archaeological site. A total of 71 C_4_ species (47 annual and 24 perennial) has been recorded across Greece [21], providing the scope for variability in δ^13^C values of available browse and graze. C_4_ plants may have been available seasonally inside the settlement enclosure, as suggested by the presence in the archaeobotanical assemblage of *Cynodon dactylon*, which may have grown as an arable weed on fields [22].

Along the coast, an alluvial plain consisting of unconsolidated Holocene alluvial deposits stretches for c.45 km south of the site and forms part of a zone of Quaternary alluvium and lacustrine formations that reaches up to the foot of the Pieria and Olympus mountains. The mountains are composed of Mesozoic (Lower Cretaceous, Jurassic and Triassic) limestones (to the south), and Neopaleozoic-Jurassic marbles, ophiolites and schists and Paleozoic gneisses and schists (to the west). The area between this oldest Pre-Alpine zone and the archaeological site (c.30 km) is mostly composed of Neogene formations consisting of sandstones, mudstones, clays and marls. Directly to the west of the Neogene zone is an area characterized by Lower Cretaceous flysch and ophiolites interspersed with areas of Cretaceous limestones [23–27].

**Fig 4:**
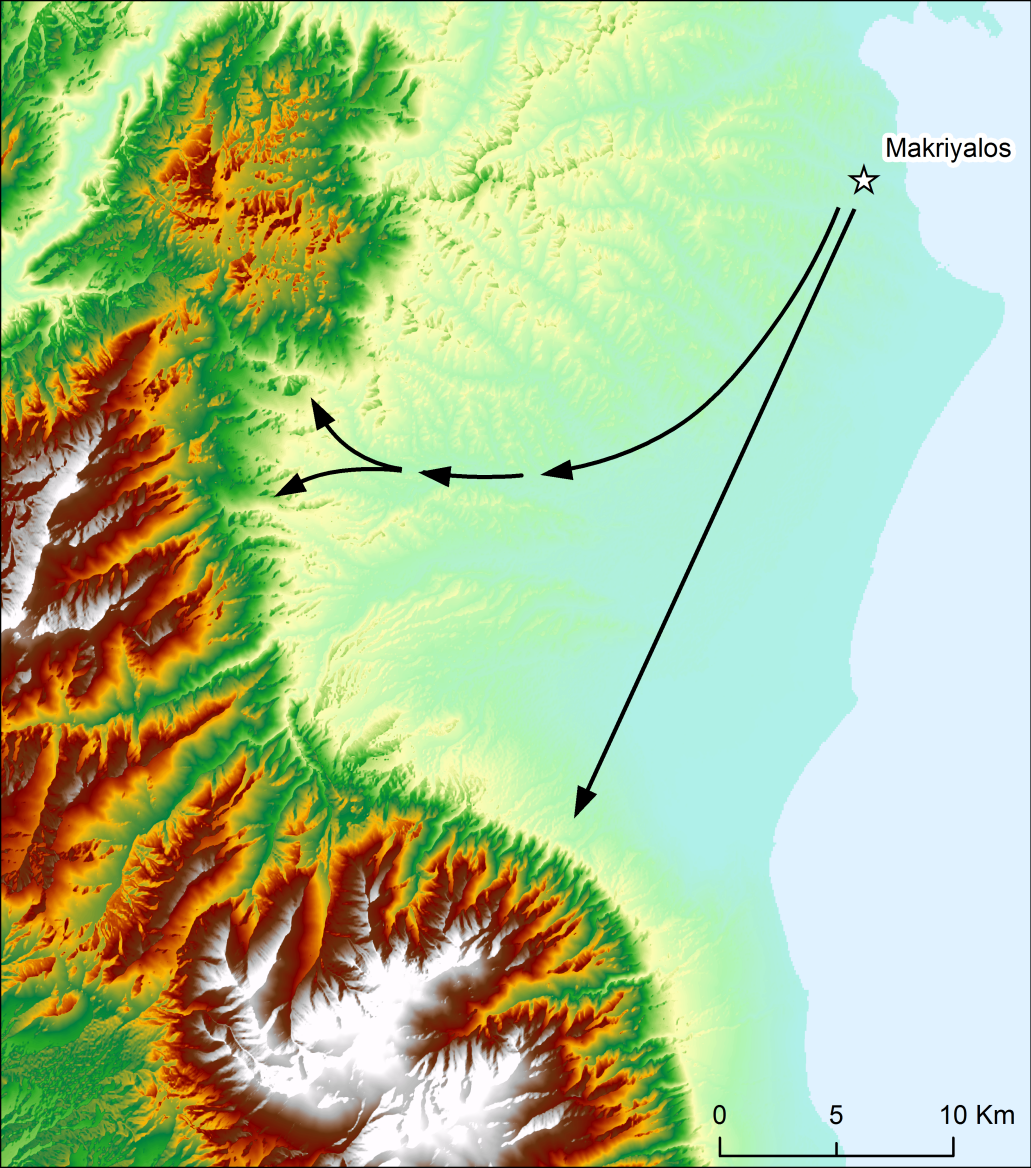
Topographical map of northern Pieria. Indicated are approximate routes, involving minimal vertical travel, from Makriyalos to geologically older zones. The northern route leads to the Cretaceous limestones and Neopaleozoic/Paleozoic formations in the Pieria Mountains. The southern route leads to the Mesozoic limestones in the Olympus mountain range. Prepared in ArcGIS 10.2 using Digital Elevation Model from Yamazaki et al. [6].

The shortest distances from Makriyalos to the main geological formations (disregarding terrain) are 19 km to the Cretaceous limestones to the west (altitude of c.300 masl), 20 km to the Lower Cretaceous flysch and ophiolites (altitude of c.500 masl), 34 km to the Paleozoic gneisses and schists (altitude of c.1500 masl) and 32 km to the Triassic limestones to the south (altitude of c.300 masl). Fig 4 shows the topography of the region and indicates the access routes, involving minimal vertical travel, from the archaeological site to the geologically older zones. Most of the suggested journeys would take place at elevations of around 200 m and only the last 4–5 km would involve ascent to altitudes over 1000 m.

## Principles of isotope analysis

The isotopic composition of archaeological bones and teeth provides information about past environmental conditions and animals’ dietary habits [28]. Stable carbon and nitrogen isotope values (δ^13^C and δ^15^N) of bone collagen reflect the photosynthetic adaptation and growing conditions of plants consumed by animals over the long-term [29,30]. Stable carbon and oxygen (δ^13^C and δ^18^O) values of tooth enamel reflect the composition of diet and the hydrological conditions experienced by the animals [31,32]. Strontium isotope ratios (^87^Sr/^86^Sr) of tooth enamel reflect the geological substrates on which the animals lived when their teeth were mineralizing, and may thus be used to assess whether or not they were raised locally to the site where they were buried [33,34].

By convention, δ^13^C and δ^15^N values are reported relative to Vienna Pee Dee Belemnite (VPDB) and ambient air (AIR), respectively, and are calculated using the following equation [35]:

$$\delta^{13}C=\frac{{}^{13}C/{}^{12}C_{sample}-{}^{13}C/{}^{12}C_{standard}}{{}^{13}C/{}^{12}C_{standard}}$$

δ^18^O values are measured relative to Vienna Standard Mean Ocean Water (VSMOW) and in this study, δ^18^O_VSMOW_ values were converted to δ^18^O_VPDB_ using the following equation [36]:

$$\delta^{18}O_{VPDB}=\frac{\delta^{18}O_{VSMOW}-30.91}{1.03091}$$

δ^13^C values of plants are mainly determined by their photosynthetic pathway [37,38]. C_3_ plants include crop species such as wheat (*Triticum*) and lentils (*Lens*) and have values ranging from -21 to -34‰, with an average of c. -27‰ [38]. C_4_ plants include arid-adapted grasses and have values ranging from -9 to -17‰, with an average around -12‰ [38]. More subtle differences in δ^13^C values of plants are caused by factors such as humidity, temperature, light and air pressure [39–42] and can be detected in the tissues of higher trophic-level consumers that form incrementally over the course of an annual cycle (for example, in the enamel of second molars). These intra-annual variations can be used to assess the seasonal dietary and mobility patterns of animals [31,43].

δ^15^N values of plants are mainly determined by the nature of the soils in which they grow and the manner in which they obtain nitrogen [44,45]. N_2_-fixing plants such as members of the *Leguminosae* family acquire N through symbiotic relationship with mycorrhiza fungi and typically have values around 0‰. These plants, and to a larger degree non-N_2_-fixing plants such as cereals, can exhibit enrichment in ^15^N due to both environmental factors (such as moisture, topography, and denitrification) and anthropogenic factors (such as the application of organic manure) [46–50].

Animals incorporate carbon and nitrogen into their tissues from the plants that they eat, and this generally results in isotopic enrichment. Bone collagen δ^15^N tends to be about 3–6‰ higher than the diet [29,51]. Diet to tissue offset in δ^13^C values is 4–6‰ for collagen and 9–13‰ for tooth bioapatite [52–56].

δ^18^O values in herbivore tooth enamel reflect the isotopic composition of ingested water (a combination of both surface and plant leaf H_2_O), which is driven by fractionation during the hydrological cycle [57,58]. In Mediterranean-type climates, δ^18^O of precipitation fluctuates predictably, exhibiting higher δ^18^O values in the dry/warm summers and lower δ^18^O values in the cold/wet winters. In addition, precipitation in higher altitudes is depleted in ^18^O compared to precipitation in lower altitudes [35,59]. More subtle differences in δ^18^O values of plant leaves and surface water are caused by factors such as humidity, temperature, air pressure, and evapo-transpiration rate [60–62].

Enamel mineralizes in two stages along the axis of tooth growth (from the crown to the enamel root junction, or ‘erj’) [63]. Oxygen from ingested water is incorporated into the carbonate moiety of tooth enamel in equilibrium with the δ^18^O value of body water [64,65] and is not remodeled after the completion of the mineralization process [32]. The second molar (M2) of many mammalian herbivores mineralizes during the first year of life and records an entire annual feeding cycle [63,66,67]. As plants reflect seasonal trends in δ^13^C and δ^18^O values, sequential analysis of tooth enamel carbonate that formed over the course of a year allows for the reconstruction of seasonal dietary patterns of herbivores [43,68].

Strontium (Sr) is incorporated into herbivore enamel primarily from plants, which obtain this trace element from the soil and the underlying bedrock [33,69]. ^87^Sr is produced by the radioactive decay of ^87^Rb and the specific Sr isotopic composition is dependent on the age of the rock and its original ^87^Rb content. As a result, older rocks tend to have higher ^87^Sr/^86^Sr ratios than younger rocks [70]. The modern ratio of the ocean is 0.7092 and because Sr from the ocean is carried via rainfall and deposited on coastal regions, these areas can show a noticeable ‘sea spray effect’ [71]. Ratios of ^87^Sr/^86^Sr from tooth enamel can be used to identify possible outsiders–people/animals born outside the local environment where they ended up buried (e.g. [72,73]). In this context, the definition of ‘local’ depends mostly on the variability of the surrounding geology.

Establishing the variability in local Sr isotope ratios is critical for interpreting ^87^Sr/^86^Sr ratios from archaeological materials. This can be done by measuring isotope ratios of bioavailable Sr from samples of modern biological materials (such as ground vegetation, tree leaves, snail shells, water and soil leachates) from different geological zones in the surrounding landscape [34,74–76]. Each location will have a range of ^87^Sr/^86^Sr ratios, rather than a single number, due to inherent variability in different rock-mineral compositions, plant parts and inputs from different water sources [77].

## Materials and methods

### Stage 1: Bulk bone collagen and charred plant δ^13^C and δ^15^N values

In Stage 1, 73 bulk samples of bone collagen were extracted from postcranial elements of humans (*Homo sapiens*, *n* = 12) and animals (cattle, *Bos taurus*, *n* = 19; goats, *Capra hircus*, *n* = 19; sheep, *Ovis aries*, *n* = 20, red deer, *Cervus elaphus*, *n* = 3, see Table 1). Sampling took account of side of body and size of the element (*femora* for humans, *humeri* for domestic animals and *radius/tibiae* of deer) to ensure that no individual was sampled twice. The humans sampled represent distinct individuals to those previously measured by Triantaphyllou (*n* = 18) [12]. Sheep and goat samples were differentiated on the basis of morphological characteristics following Boessneck et al. [78]. The samples came from both feasting (F) and non-feasting (NF) contexts from the first phase of occupation of Makriyalos. Fifteen additional samples of mandibular bone collagen (12 cattle, 3 sheep) were analyzed in order to guide incremental sampling of tooth crowns in Stage 2. These values will not be included in the bulk collagen analysis, as they may represent the same individuals as the postcranial samples.

**Table 1:** List of samples analyzed in Stage 1.

Common name	Latin name	*n* =
Human	*Homo sapiens*	12
Cattle	*Bos taurus*	19
Sheep	*Ovis aries*	20
Goat	*Capra hircus*	19
Red deer	*Cervus elaphus*	3
Emmer grain	*Triticum dicoccum*	5

Collagen was extracted from the bones following a modified Longin procedure [79] described in Richards and Hedges [80]. All reported bone collagen isotope measurements have C/N ratios in the range of 2.9–3.6, indicating well-preserved collagen [81].

Due to the nature of the archaeobotanical assemblage, only 5 samples of charred grains of emmer (*Triticum dicoccum*, Shrank.) were available for analysis. Each sample contained 5 whole grains (or fragments thereof) and was pre-treated using 0.5 M HCl for 30 min at 80°C [82]. The plant measurements were corrected for the charring offset of 0.3‰ (for δ^15^N values) and 0.1‰ (for δ^13^C values) following experimental findings by Nitsch et al. [83].

Styring et al. [84] reported the average values of the bone collagen and three emmer grain δ^13^C and δ^15^N values obtained from the first set of measurements (RF, see S1 File), but this is the first time that the full dataset (including duplicate measurements and excluding two human samples re-identified as cattle) is presented and analyzed contextually.

### Stage 2: Incremental tooth enamel carbonate δ^13^C and δ^18^O values

In Stage 2, incremental samples of tooth enamel were obtained from 8 cattle and 4 sheep second molars (M2) extracted from almost-complete mandibles recovered from feasting and non-feasting (habitation) contexts. The use of mandibles (as opposed to loose teeth) ensured the accurate identification of the type of tooth and species. Measurement of 15 samples of mandibular collagen revealed which of the individuals had predominantly C_3_ and which had predominantly C_4_ long-term diets, and samples for this stage of analysis were chosen in an effort to have both groups represented equally.

Ages of the individuals were estimated from tooth eruption and wear following Payne [85] for sheep and following an adaptation of Payne’s method for cattle [86,87]. Individuals ranged in age between older juveniles and adults (see Table 2 for sample descriptions). The tooth-crown heights of the teeth ranged between 42–61 mm for cattle and 26–32 mm for sheep.

**Table 2:** Descriptions of cattle and sheep teeth sampled in Stage 2. Eruption/wear stages and approximate ages were assigned following Payne [85] for sheep and Halstead [86] and Jones and Sadler [87] for cattle.

ID	species	context	deposit type	crown height (in mm)	number of sub-samples	wear stage	estimated age
MKS001	cattle	R 0654080	habitation	42	18	EF	2–3.5 years
MKS004	cattle	Xi 0121014	habitation	55	22	E	2–3 years
MKS006	cattle	R 0642017	habitation	52	26	E	2–3 years
MKS007	cattle	R 0543090	habitation	61	32	D	16–28 months
MKS014	cattle	Pi 0572031	feasting	54	22	D	16–28 months
MKS015	cattle	Pi 0573058	feasting	53	22	D	16–28 months
MKS016	cattle	Pi 0564018	feasting	58	27	E	2–3 years
MKS017	cattle	Pi 0572031	feasting	55	23	D	16–28 months
MKS104	sheep	R 0642008	habitation	26	10	G	4–6 years
MKS105	sheep	Pi 0572012	feasting	31	13	E	2–3 years
MKS106	sheep	Pi 0571035	feasting	27	13	F	3–4 years
MKS107	sheep	O 0672022	habitation	32	15	E	2–3 years

**Fig 5:**
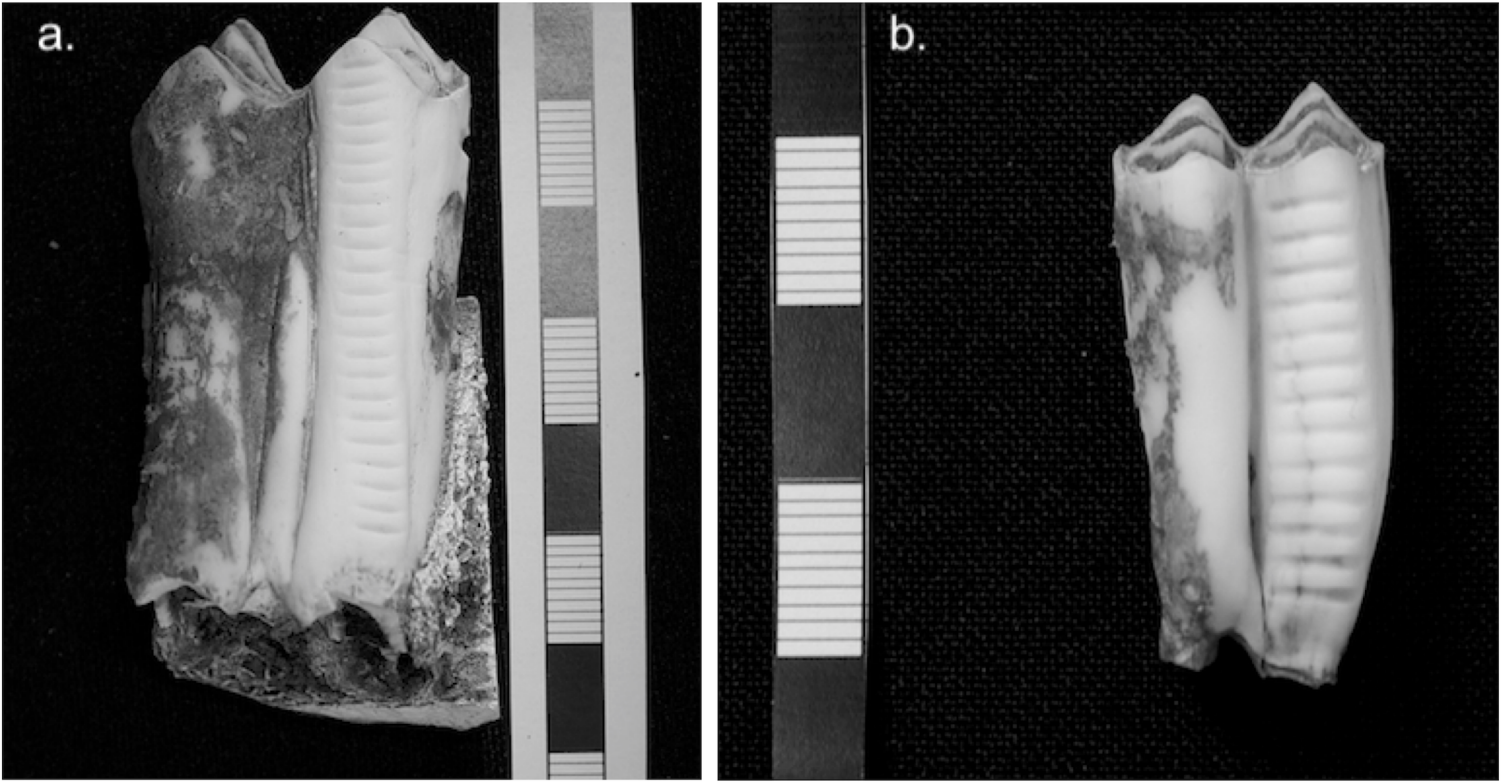
Photographs showing the sampling procedure in Stage 2. The images show the sequences of enamel sub-samples obtained by drilling from the buccal side of herbivore second molars. (a) cattle, (b) sheep.

Enamel surfaces were cleaned with a laboratory sandblaster (aluminium oxide) and the external surface was removed by drilling with a tungsten drill bit. Powdered enamel samples weighing 5–10 mg were taken approximately every 1 mm along the growth axis on the buccal side of each tooth, starting at the cusp and ending at the enamel root junction (erj), following procedures outlined in Balasse [88]. See Fig 5A–5B for photographs of teeth after the completion of incremental sampling. The number of sub-samples ranged from 18–32 (cattle) to 10–15 (sheep) per tooth, amounting to 192 (cattle) and 51 (sheep) in total.

All enamel samples were pre-treated using Ca–buffered 1 M acetic acid for 30 min at room temperature to remove any exogenous carbonates [89]. Fourier Transform Infrared Spectra (FTIR) of two random samples (cattle, MKS015; sheep, MKS104) showed that the teeth were not contaminated with exogenous calcite (which has a peak at 1436 cm^-1^) and had not undergone significant re-crystallization (the splitting factor of modern enamel is c.4.1 (p.291) [90], and the splitting factors of the measured teeth were MKS015 = 3.9, MKS104 = 4.0; see S1 and S2 Figs).

### Stage 3: Modern plant and archaeological tooth enamel ^87^Sr/^86^Sr ratios

**Fig 6:**
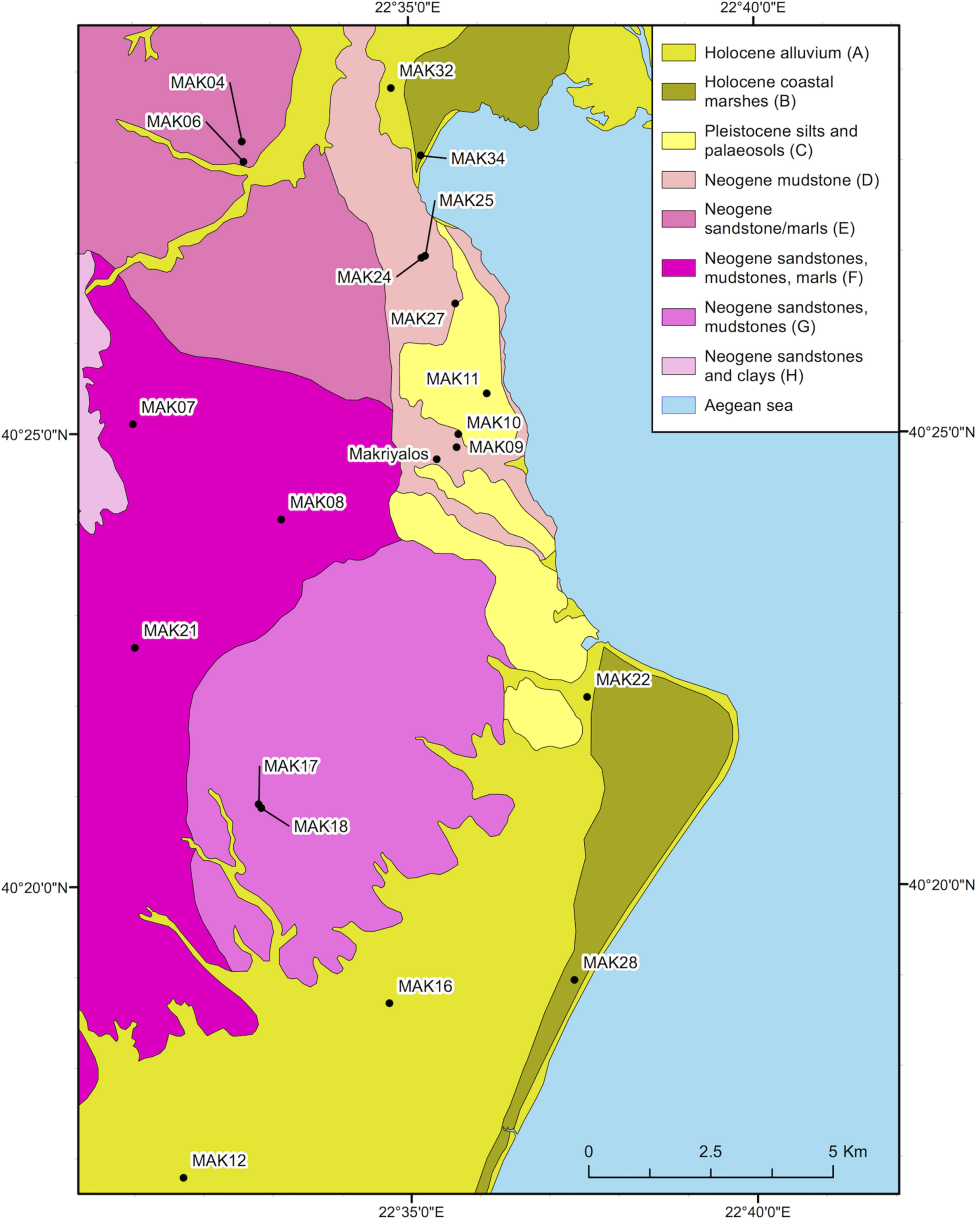
Geological map of the immediate environment around Makriyalos, showing the sampling locations of modern vegetation used to establish the local range of bioavailable strontium. The samples were taken in a radius of 15 km within the archaeological site. The map was prepared using information from the Greek Institute of Geology and Mineral Exploration [24]. The published information was digitized using ArcGIS 10.2 and geo-referenced to the Greek grid.

To establish the isotopic range of bioavailable Sr in the region around Makriyalos, samples of modern vegetation with variable depths of rooting systems (tree leaves and ground vegetation) were obtained at 34 sampling locations across the seven geological formations within c.15 km of the archaeological site (see Fig 6). 20 samples were submitted for ^87^Sr/^86^Sr ratio measurement. MAK22 was collected as close as possible to the coastal marshes (Zone B) and even though it lies on the border between this zone and the Holocene alluvium (Zone A), it will be grouped here with the other Zone B samples due to its proximity to this latter zone.

The plant samples were collected into dry paper bags and air dried before being transported to the laboratory for microwave-assisted plant digestion (protocol based on a modified Heier et al. [91] technique, [75], see S1 File).

**Fig 7:**
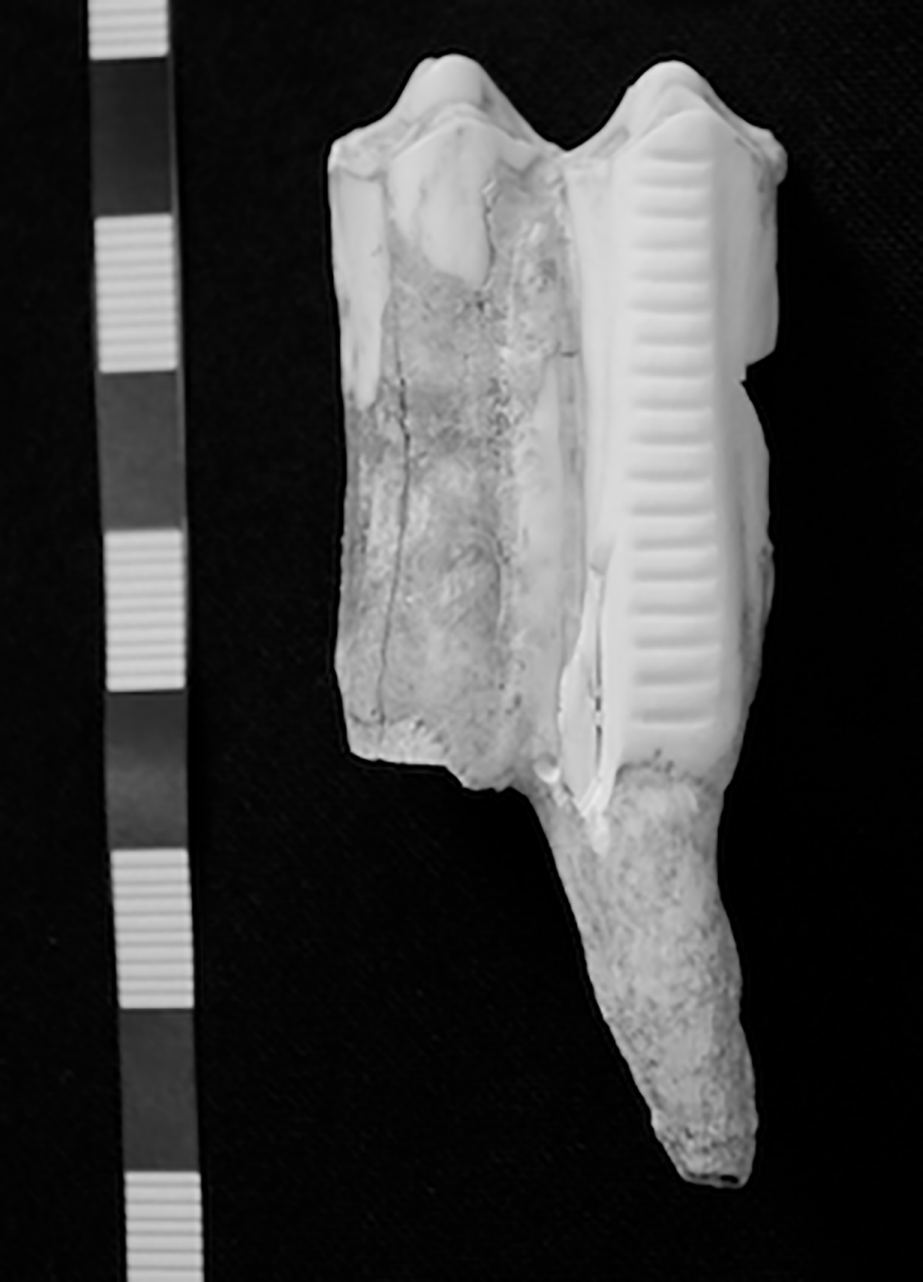
Photograph showing the sampling procedure in Stage 3. Image shows a cattle tooth (sample MKS001) after the completion of both incremental sampling for enamel carbonate stable isotope analysis (18 sub-samples) and removal of 2 sub-samples for ^87^Sr/^86^Sr ratio measurement (one sample from the bottom left of the cleaned area, one sample from the middle right of the cleaned area).

The cattle enamel samples (weighing 10–20 mg) came from the same teeth as were previously analyzed in Stage 2. Two sub-samples were removed from each tooth using a Dremel tool with a diamond drill bit: one from the portion that mineralized in the winter (where δ^18^O value was at its minimum) and one from the portion that mineralized in the summer (where δ^18^O value was at its maximum; total *n* = 16). See S1 File for details of chemical pre-treatment and Fig 7 for photographs of the incisions created through drilling.

Permission to carry out the scientific analyses of the materials from Makriyalos was issued by the Ephorate of Pieria, Hellenic Ministry of Education and Religious Affairs, Culture and Sports (permit YΠΠO/ΣYNT/Φ44/1118/27399–11–03–2008). The samples are stored in a government-controlled storage facility in modern-day Makriyalos, Greece, and are not publically accessible. For research purposes, access to the material can be sought from the Greek Ministry of Culture, with support from the excavation co-director (Dr M Pappa). The study complied with all relevant regulations.

## Results

### Stage 1: Bulk bone collagen and charred plants

**Fig 8:**
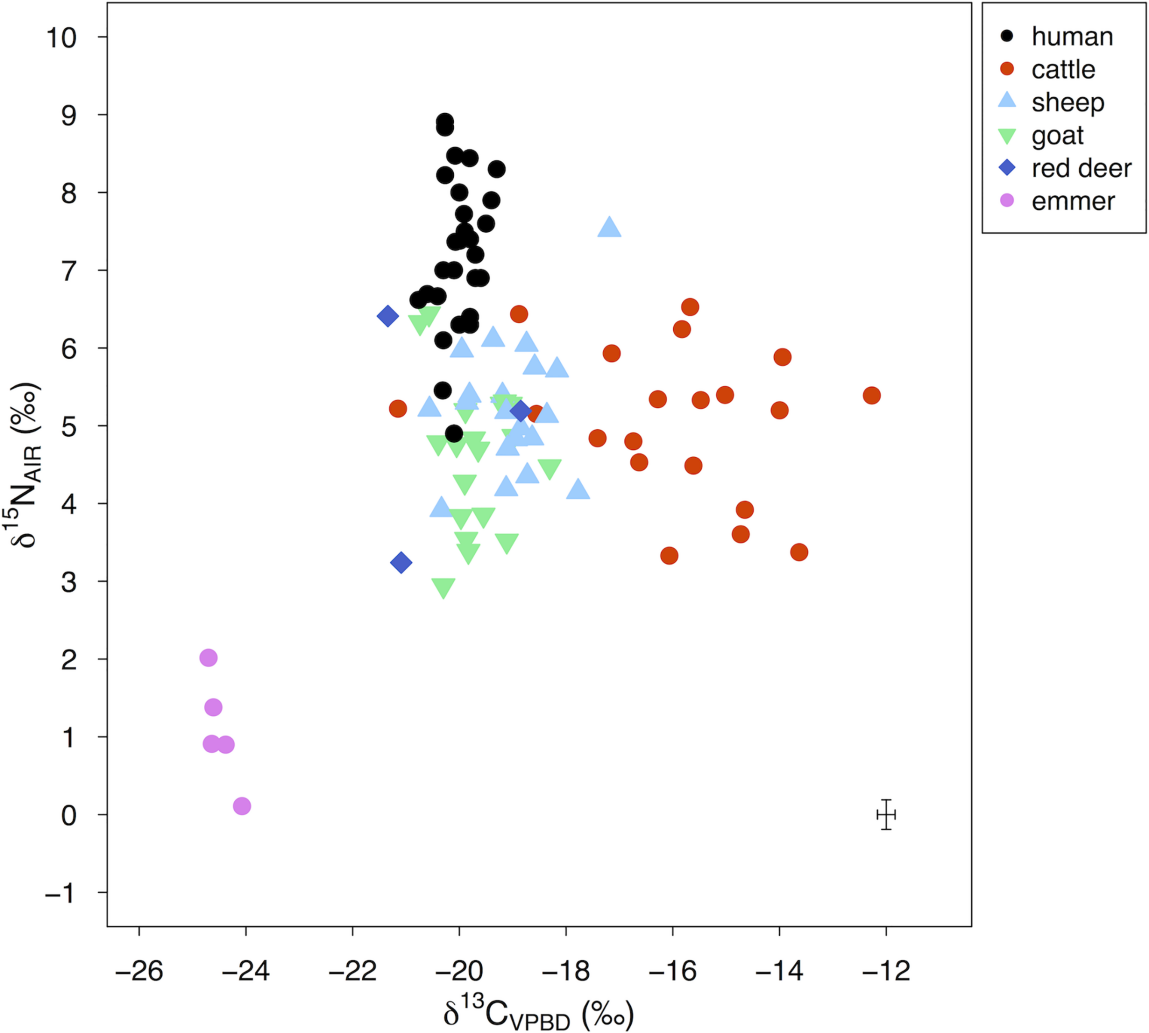
δ^13^C and δ^15^N values of human and domestic animal bone collagen and charred emmer grain from Makriyalos I. Measurement error is shown in the bottom-right corner of the plot. Included are also previous measurements of contemporary human samples (*n* = 18) [12]. Summary statistics of three out of the five emmer grains presented in this figure have previously been published in [84].

Fig 8 shows the δ^13^C and δ^15^N values of human/animal bone collagen and emmer grain measured in this study alongside the values of Makriyalos I humans measured previously (*n* = 18) [12]. Summary statistics of all new measurements are shown in Table 3. Contextual information is detailed in S1 Table and individual measurements are presented in S2 Table.

**Table 3:** Summary statistics of δ^13^C and δ^15^N values of bone collagen and charred grain from Makriyalos I. δ^13^C values are reported relative to VPDB, δ^15^N values relative to AIR. Summary statistics of three out of the five emmer grains presented in this table have previously been published in [84].

species	*n* =	δ^15^N_max_	δ^15^N_min_	δ^15^N_mean_	δ^15^N_SD_	δ^15^N_range_	δ^13^C_max_	δ^13^C_min_	δ^13^C_mean_	δ^13^C_SD_	δ^13^C_range_
cattle	19	6.5	3.3	5.1	1.0	3.2	-12.3	-21.3	-16.0	2.1	8.9
sheep	20	7.5	3.9	5.2	0.8	3.6	-17.2	-20.6	-19.0	0.8	3.4
goat	19	6.4	2.9	4.6	0.9	3.5	-18.3	-20.7	-19.7	0.6	2.4
red deer	3	6.4	3.2	4.9	1.6	3.2	-18.9	-21.3	-20.4	1.4	2.5
human	12	8.9	5.5	7.6	1.1	3.5	-19.8	-20.8	-20.2	0.3	1.0
emmer	5	2.0	0.1	1.1	0.7	1.9	-24.1	-24.7	-24.5	0.3	0.6

Cattle exhibit higher δ^13^C values than the humans and other animals (see Table 3). The non-parametric Kruskal-Wallis test reveals significant differences between the δ^13^C means of the five groups (*H* (4) = 43.6, *p* < 0.01; the non-parametric test was used because the Shapiro-Wilk test showed that the data is not normally distributed, *W* = 0.87, *p* < 0.01). A post-hoc Bonferroni test indicates that the significant differences are between the cattle and each of the other species (*p* < 0.01 for all four pairs), but not between the pairs of the other species.

Human δ^15^N values are higher compared to all the other animal species. The mean δ^15^N values of the five groups are statistically different (ANOVA test, *F* (4,69) = 19.28, *p* < 0.01; ANOVA was used because the Shapiro-Wilk test showed that the data is normally distributed, *W* = 0.95, *p* < 0.01, and Levene’s test showed that the variance is homogenous, *F* (4,69) = 0.79, *p* = 0.53). A post-hoc Bonferroni test specifies that the differences are between humans and each of the other species (*p* < 0.01 for all four pairs), but not between the pairs of the other species.

Triantaphyllou [12] previously measured human bone collagen δ^13^C and δ^15^N values from Makriyalos I (*n* = 18) as well as domestic pig (*n* = 5), wild boar (*n* = 5) and red deer (*n* = 5) from Makriyalos II. The previous human δ^13^C values (-19.8 ± 0.3‰) are slightly higher compared to the values measured in this study and the corresponding δ^15^N values (6.8 ± 0.9‰) are lower. The differences are statistically significant for δ^13^C values (two-tailed equal variance student’s *t*-test, *t* = 3.66, *df* = 28 *p* < 0.01), and may be the result of inter-laboratory variability. The differences in δ^15^N values are not statistically significant (two-tailed equal variance student’s *t*-test, *t* = -1.99, *df* = 28, *p* = 0.054).

Most of the domestic animals measured in this study are enriched in ^13^C compared to the wild animals and domestic pig from MKII: wild boar, *n* = 5, -20.4 ± 0.4‰; red deer, *n* = 5, -21.7 ± 0.4‰; domestic pig, *n* = 5, -20.5 ± 0.3‰ [12]. The corresponding δ^15^N values are slightly lower for wild boar (4.2 ± 0.8‰) and red deer (3.2 ± 1.3‰), but not domestic pigs (5.3 ± 0.8‰).

**Fig 9:**
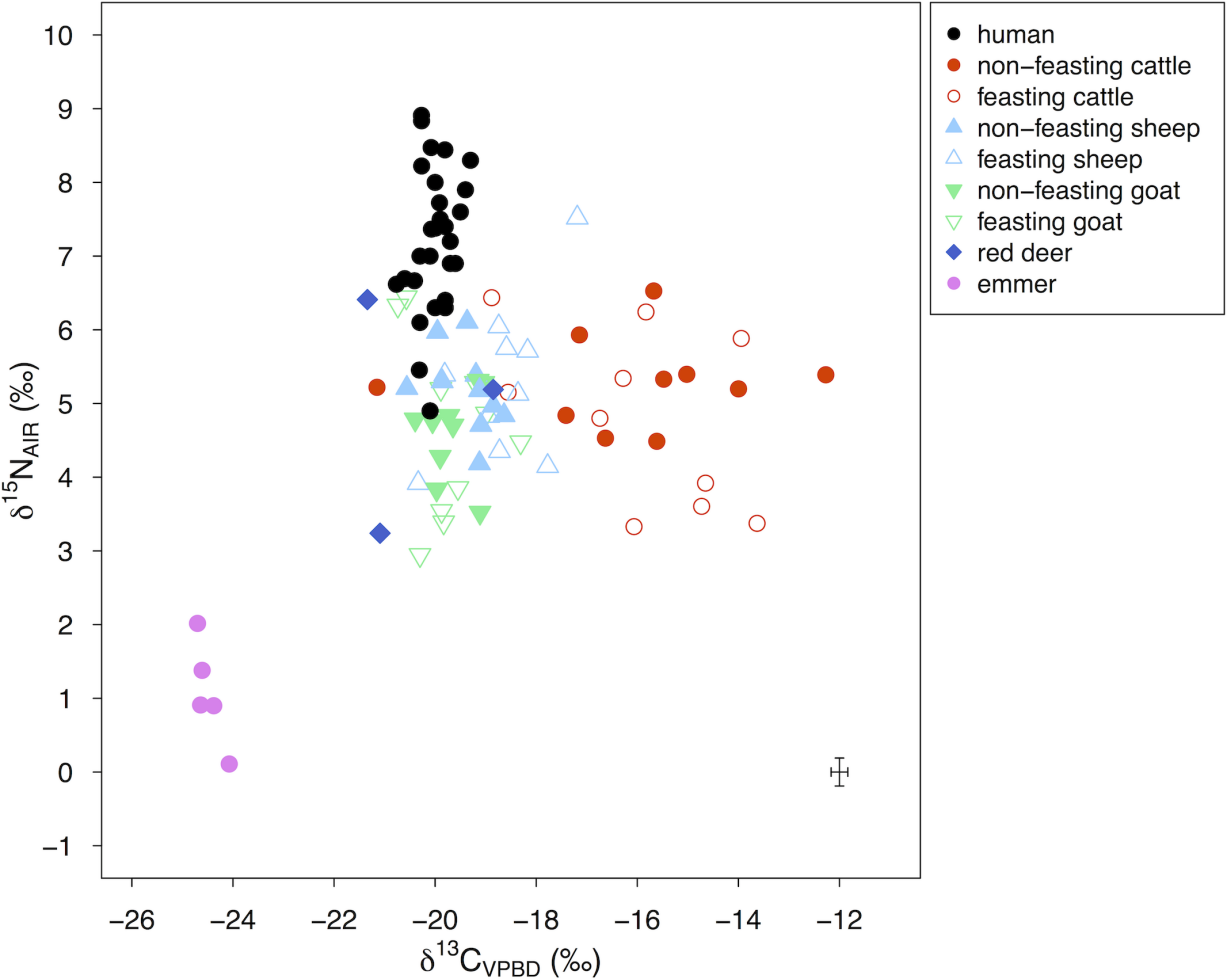
δ^13^C and δ^15^N values of bone collagen and charred emmer grain from Makriyalos I separated by context. Samples from feasting contexts (Pit 212 and Pit 214) are shown using open symbols. Samples from non-feasting contexts (habitation) are shown using filled symbols. Measurement error is shown in the bottom-right corner of the plot. Included are also previous measurements of contemporary human samples (*n* = 18) [12]. Summary statistics of three out of the five emmer grains presented in this table have previously been published in [84].

Fig 9 shows the data grouped according to context type: feasting (F, Pit 212 and 214) and non-feasting (NF, all other domestic and refuse contexts). δ^13^C values range from -18.8 to -13.6‰ for F cattle, from -21.2 to -12.3‰ for NF cattle, from -20.3 to -17.2‰ for F sheep, from -20.6 to -18.6‰ for NF sheep, from -20.7 to -18.3‰ for F goats, and -20.4 to -19.0‰ for NF goats. δ^15^N values range from 3.3 to 6.4‰ for F cattle, 4.5 to 6.5‰ for NF cattle, 3.9 to 7.5‰ for F sheep, 4.2 to 6.1‰ for NF sheep, 2.9 to 6.4‰ for F goats, and 3.5 to 5.3‰ for NF goats.

There are statistically significant differences in bone collagen δ^13^C values between animals from the feasting deposits and those from non-feasting contexts (Kruskal-Wallis test, *H* (5) = 34.34, *p* < 0.01), and a Bonferroni post-hoc test reveals that the differences exist between pairs of different animals (F cattle and F sheep, F cattle and NF sheep, NF cattle and F sheep, etc.), but not between pairs of the same animals (F cattle and NF cattle, F sheep and NF sheep, F goat and NF goat (for all 3 pairs, *p* = 1.0). There are no significant differences in δ^15^N values of all the groups (ANOVA test, *H* (5) = 6.33, *p* = 0.28).

### Stage 2: Tooth enamel carbonate

**Fig 10:**
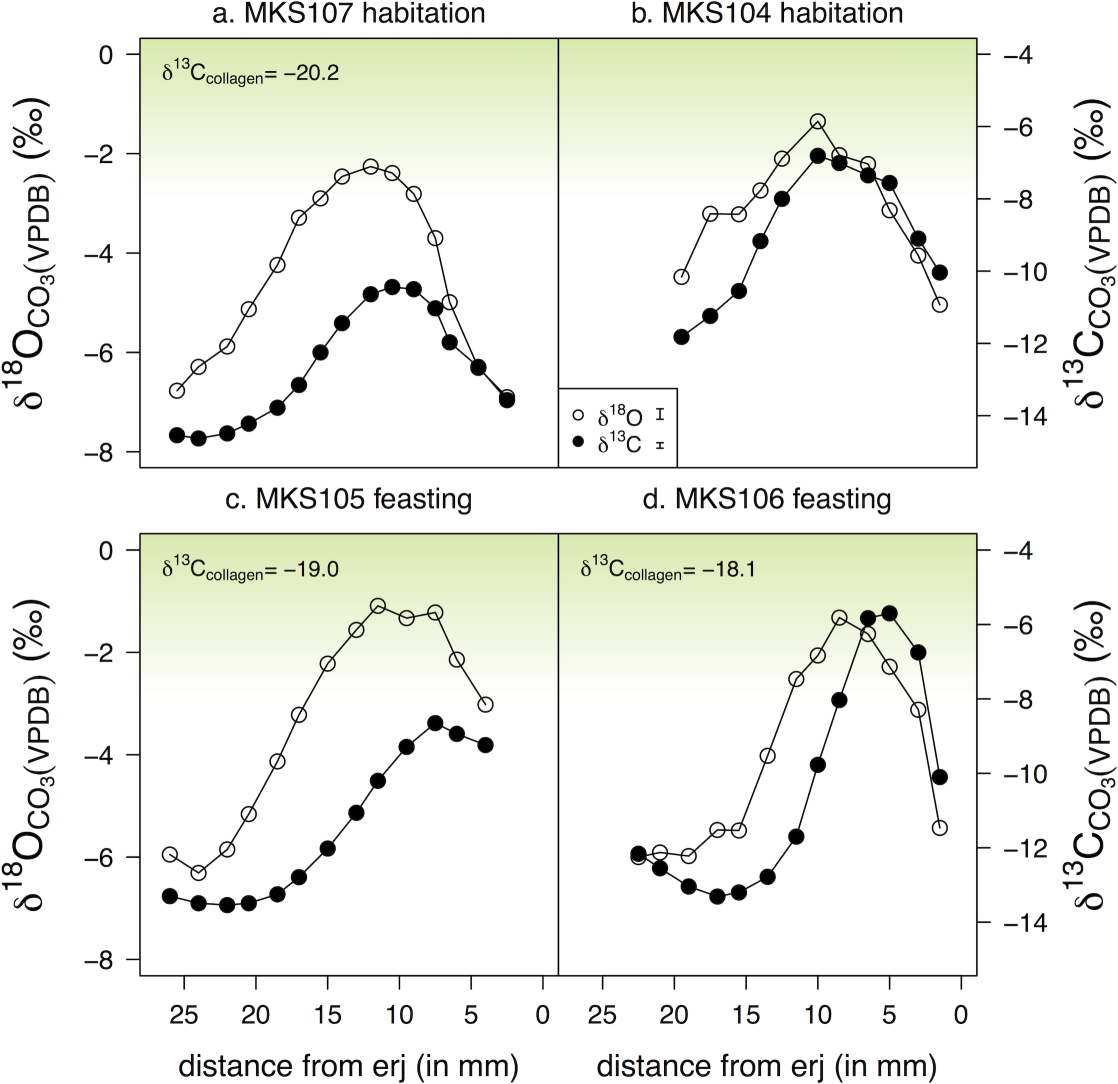
Incremental tooth enamel carbonate δ^18^O and δ^13^C values of sheep from Makriyalos I. (a–b) samples from non-feasting contexts (habitation), (c–d) samples from feasting context (Pit 212). Positions of sub-samples are recorded as distance (in mm) from the enamel root junction (erj). δ^13^C values of matching mandibular collagen (where C/N ratio was acceptable) are included for reference. Green shading indicates theoretical values of consumers with increasing amounts of C_4_ vegetation in their diet (the lower endpoint is set at -8‰ [38]). Measurement error for each element is shown in the legend.

**Fig 11:**
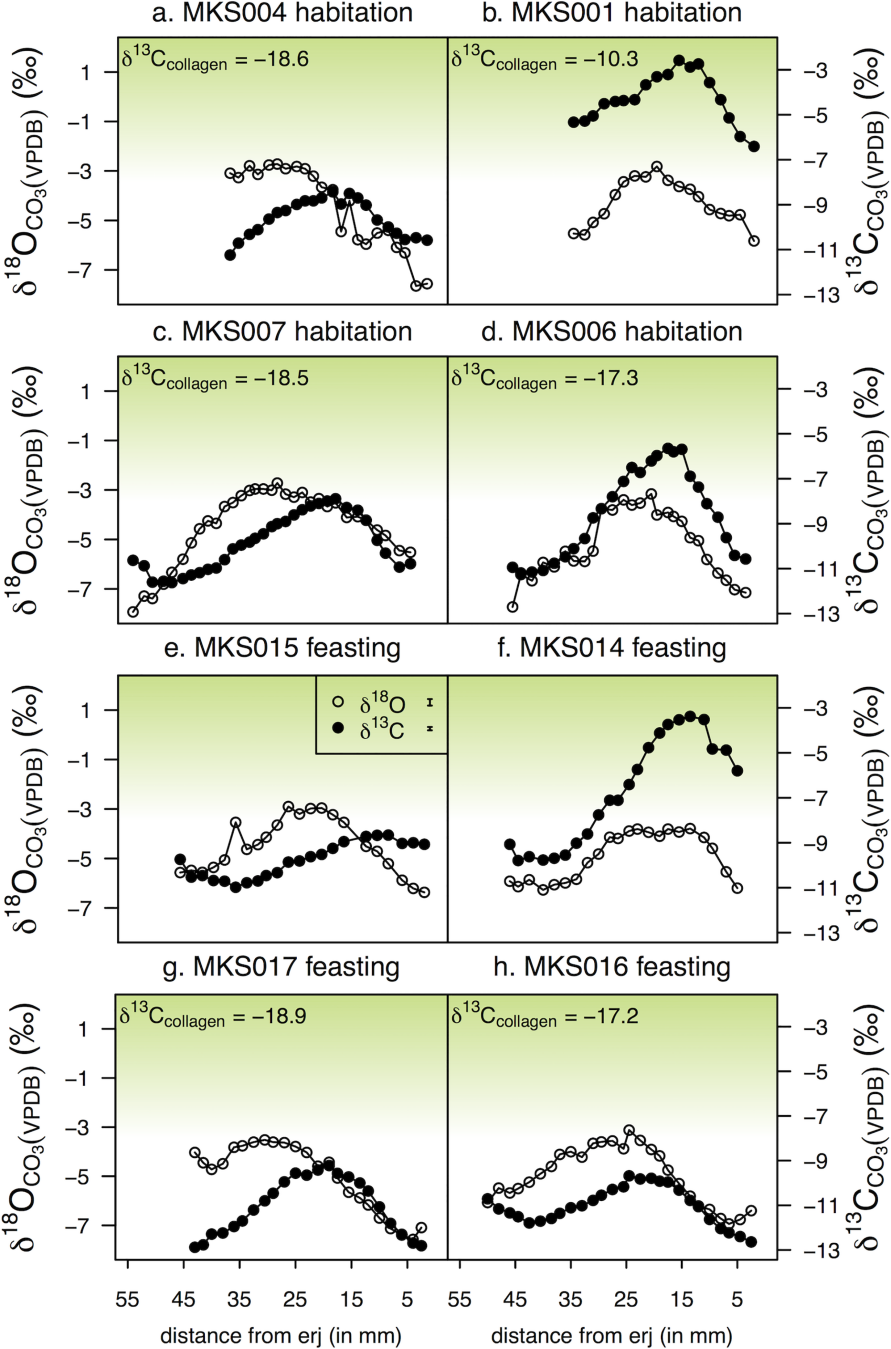
Incremental tooth enamel carbonate δ^18^O and δ^13^C values of cattle from Makriyalos I. (a–d) samples from non-feasting contexts (habitation), (e–h) samples from feasting context (Pit 212). Positions of sub-samples are recorded as distance (in mm) from the enamel root junction (erj). δ^13^C values of matching mandibular collagen (where C/N ratio was acceptable) are included for reference. Green shading indicates theoretical values of consumers with increasing amounts of C_4_ vegetation in their diet (the lower endpoint is set at -8‰ [38]). Measurement error for each element is shown in the legend.

Incremental tooth enamel carbonate δ^13^C and δ^18^O values are shown in Fig 10 (sheep) and Fig 11 (cattle). Table 4 shows the summary statistics and S3 Table presents the matching mandibular collagen and tooth enamel values for each individual. Individual measurements are presented in S4 Table (cattle) and S5 Table (sheep).

**Table 4:** Summary statistics of incremental tooth enamel carbonate δ^13^C and δ^18^O values from Makriyalos I. Both δ^13^C and δ^18^O values are reported relative to VPDB. Δ values indicate the amplitude of intra-tooth variation.

Tooth ID	Max δ^13^C (‰)	Min δ^13^C (‰)	Δ^13^C (‰)	Max δ^18^O (‰)	Min δ^18^O (‰)	Δ^13^O (‰)	r^2^*	
**cattle**								
MKS001	-2.6	-6.4	3.8	-2.8	-5.8	3.0	0.55	
MKS004	-8.4	-11.2	2.8	-2.7	-7.7	4.9	0.06	
MKS006	-5.7	-11.2	5.5	-3.2	-7.7	4.6	0.72	
MKS007	-7.9	-11.6	3.7	-2.7	-7.9	5.2	0.48	
MKS014	-3.4	-9.8	6.4	-3.8	-6.3	2.5	0.60	
MKS015	-8.7	-11.0	2.3	-2.9	-6.4	3.5	0.01	
MKS016	-9.7	-12.6	3.0	-3.1	-6.9	3.8	0.54	
MKS017	-9.2	-12.9	3.7	-3.5	-7.6	4.1	0.09	
sheep								
MKS104	-6.8	-11.8	5.0	-1.4	-5.0	3.7	0.55	
MKS105	-8.7	-13.5	4.9	-1.1	-6.3	5.2	0.68	
MKS106	-5.7	-13.3	7.6	-1.3	-6.0	4.7	0.58	
MKS107	-10.4	-14.6	4.2	-2.3	-6.9	4.6	0.62	

* Correlation coefficient for the linear relationship between δ^13^C and δ^18^O values in each tooth

δ^13^C values vary from -14.6 to -5.7‰ in sheep and from -12.9 to -2.6‰ in cattle, with mean amplitude of intra-tooth variation at 5.4‰ (from 4.2 to 7.6‰) for sheep and at 3.9‰ (from 2.3 to 6.4‰) for cattle. δ^18^O values vary from -6.9 to -1.1‰ in sheep and -7.9 to -2.7‰ in cattle, with mean intra-tooth variation of 4.6‰ (from 3.7 to 5.2‰) for sheep and 3.9‰ (from 2.5 to 5.2‰) for cattle.

Three of the four sheep yielded mandibular collagen with acceptable C/N ratios and the δ^13^C values suggest that these individuals had a small input of C_4_ vegetation in their long-term diet (see S3 Table). In terms of their early-life diets, two of the four sheep analyzed (MKS104 and MKS106) exhibit small C_4_-plant contributions during the summer, with δ^13^C values above -8‰ coinciding with their maximum δ^18^O values. These individuals were slaughtered at older ages (MKS104: 4–6 years, MKS106: 3–4 years) than the other two sheep (both died at 2–3 years of age; see Table 2).

Six out of eight cattle samples yielded mandibular collagen with acceptable C/N ratios and their δ^13^C values (ranging from -10.3 to -18.9‰) indicate varying inputs of C_4_ plants in these animals’ long-term diets. The tooth enamel carbonate values of three individuals (MKS001, MKS006, MKS014) show notable contribution of C_4_ plants during the summer, while the rest of the individuals (MKS004, MKS007, MKS015, MKS016, and MKS) exhibit C_3_-plant based diets during the first year of their lives. The cattle were all killed in a narrower timeframe than the sheep (either between 16 and 28 months or between 2 and 3/3.5 years), so no distinctions can be made relative to the age at which the animals were slaughtered.

The correlation between the δ^13^C and δ^18^O values is similar in the case of sheep (for linear regressions between matching δ^13^C and δ^18^O sequences, r^2^ values are 0.55 for MKS104, 0.68 for MKS105, 0.58 for MKS106, and 0.62 for MKS107). In the case of cattle, five individuals exhibit correlation between δ^13^C and δ^18^O values (r^2^ = 0.55 for MKS001, 0.72 for MKS006, 0.54 for MKS016, 0.60 for MKS014 and 0.48 for MKS007), while three individuals do not (r^2^ = 0.06 for MKS004, 0.09 for MKS017 and 0.01 for MKS015).

There are no systematic differences between individuals discarded in the feasting deposit and those discarded in non-feasting contexts, i.e. it is not the case that feasting cattle had significantly distinct diets compared to non-feasting cattle.

### Stage 3: Modern plants and archaeological cattle teeth

**Fig 12:**
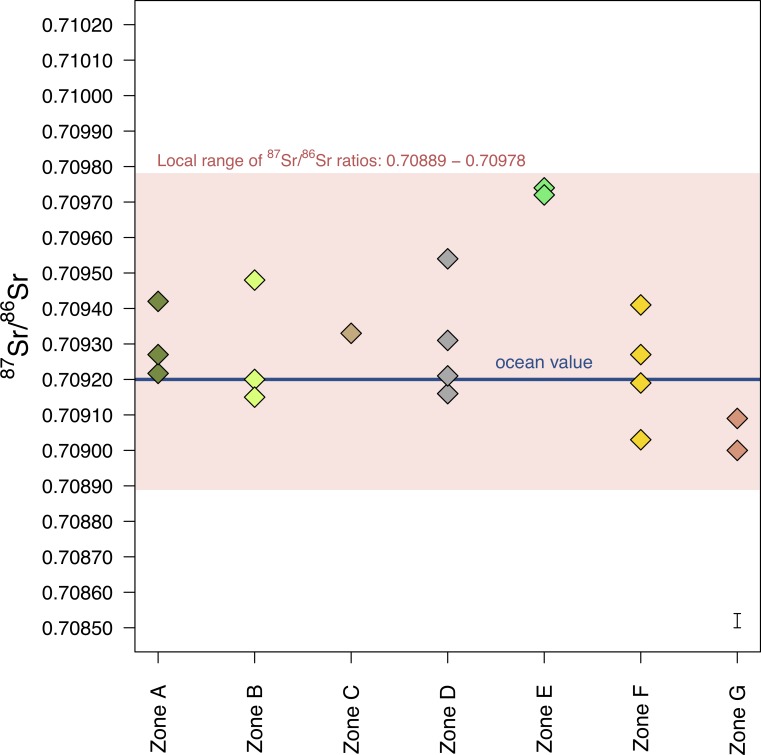
^87^Sr/^86^Sr ratios of modern vegetation from coastal northern Pieria. The samples were collected from seven geological zones within 15 km of the archaeological site. The measurements are used to establish the ‘local range’ of ^87^Sr/^86^Sr ratios. For descriptions of the zones and location of sampling points, see Fig 6. Measurement error is shown in the bottom-right of the plot.

Fig 12 shows the ^87^Sr/^86^Sr ratios of the modern vegetation in the seven geological zones around Makriyalos (raw data presented in S6 Table). The results show that apart from higher ratios in Zone E (Neogene sandstones and marls), all the zones in the local environment–Neogene to Holocene in age–have indistinguishable ^87^Sr/^86^Sr ratios. The higher ratios in Zone E may reflect an alluvial component originating in a more radiogenic source. The ratios of all plants range between 0.70900–0.70974, with mean ratio of 0.70929 ± 0.00041 (2σ). As the mean ± 2σ interval (0.70889–0.70970) does not include the ratios from Zone E (which reaches very close to the site; ratios of 0.70974 and 0.70972), in order to define the ‘local range’ of ^87^Sr/^86^Sr ratios, the top boundary was extended to include ratios from Zone E plus measurement error of 0.00004. Thus, the local range of ^87^Sr/^86^Sr ratios is here defined as 0.70889–0.70978.

**Fig 13:**
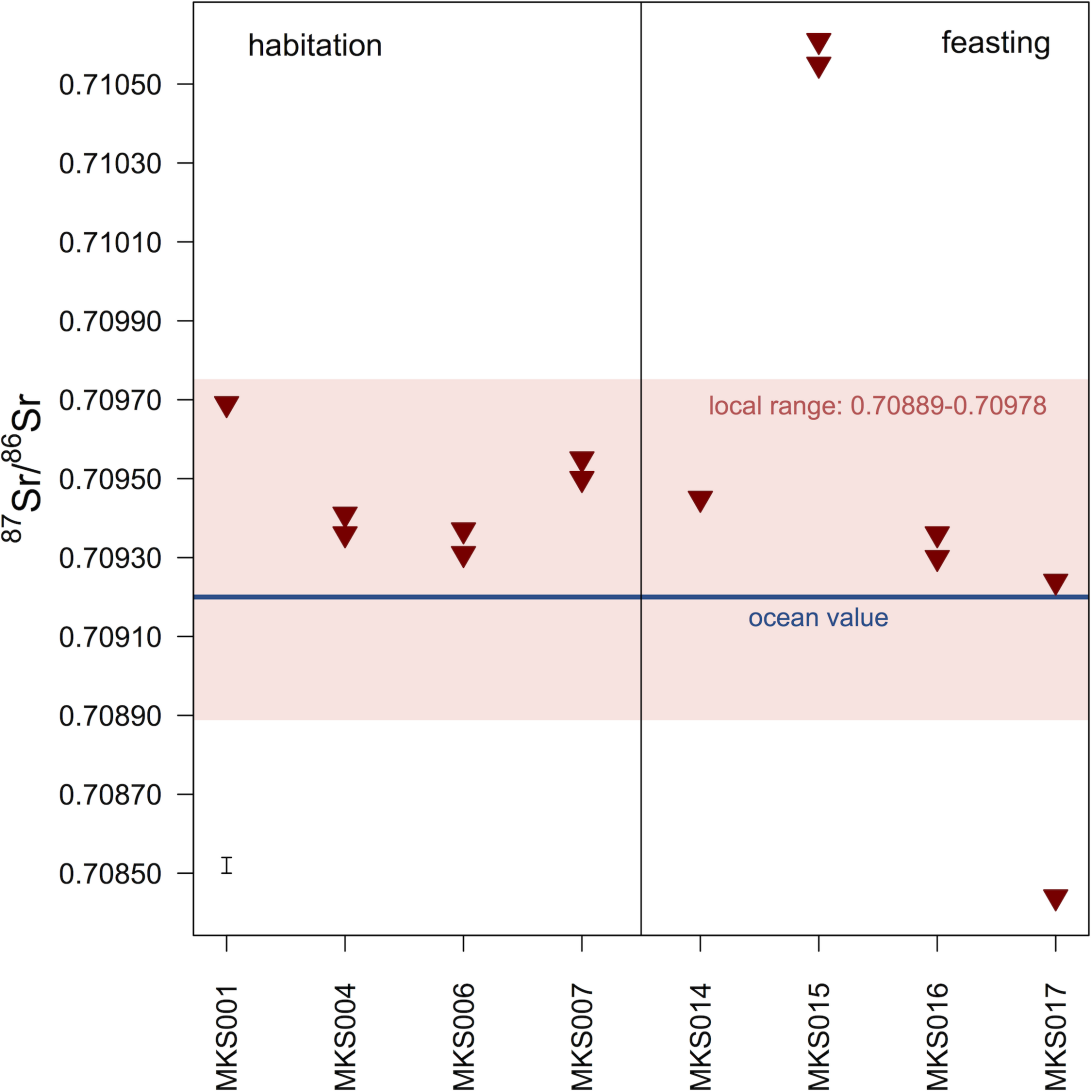
Archaeological tooth enamel ^87^Sr/^86^Sr ratios from Makriyalos. All samples were previously measured in Stage 2. Each tooth was sampled twice: 1) in the region that mineralized during the summer and 2) in the region that mineralized during the winter (determined using the δ^18^O_max_ and δ^18^O_min_ values, respectively). Measurement error is shown in the bottom-left corner of the plot.

Fig 13 and Table 5 show the ^87^Sr/^86^Sr ratios of archaeological cattle enamel. Two individuals (MKS001, habitation, and MKS014, feasting) have identical summer and winter ratios, five individuals (MKS004, MKS006, MKS007, MKS015, MKS016) have summer and winter ratios within 0.00006 of each other, and one individual (MKS017, feasting) has distinctly different summer and winter ratios (difference of 0.0008).

**Table 5:** ^87^Sr/^86^Sr ratios of archaeological tooth enamel from Makriyalos. 2σ uncertainty of the ^87^Sr/^86^Sr measurements is 0.00004. erj = enamel root junction.

ID	context	estimated age at death	sample position (in mm from erj)	^87^Sr/^86^Sr	Sr concentration (ppm)
MKS001–I	habitation	2–3.5 years	17.5	0.70969	70
MKS001–II	habitation	2–3.5 years	2.0	0.70969	71
MKS004–I	habitation	2–3 years	30.0	0.70941	267
MKS004–II	habitation	2–3 years	1.5	0.70936	295
MKS006–I	habitation	2–3 years	15.0	0.70931	214
MKS006–II	habitation	2–3 years	3.5	0.70937	230
MKS007–I	habitation	16–28 months	28.5	0.70950	192
MKS007–II	habitation	16–28 months	4.5	0.70955	217
MKS014–I	feasting	16–28 months	34.0	0.70945	188
MKS014–II	feasting	16–28 months	11.0	0.70945	152
MKS015–I	feasting	16–28 months	20.5	0.71061	160
MKS015–II	feasting	16–28 months	6.0	0.71055	166
MKS016–I	feasting	2–3 years	22.5	0.70930	164
MKS016–II	feasting	2–3 years	8.0	0.70936	157
MKS017–I	feasting	16–28 months	32.5	0.70844	78
MKS017–II	feasting	16–28 months	2.5	0.70924	117

**Fig 14:**
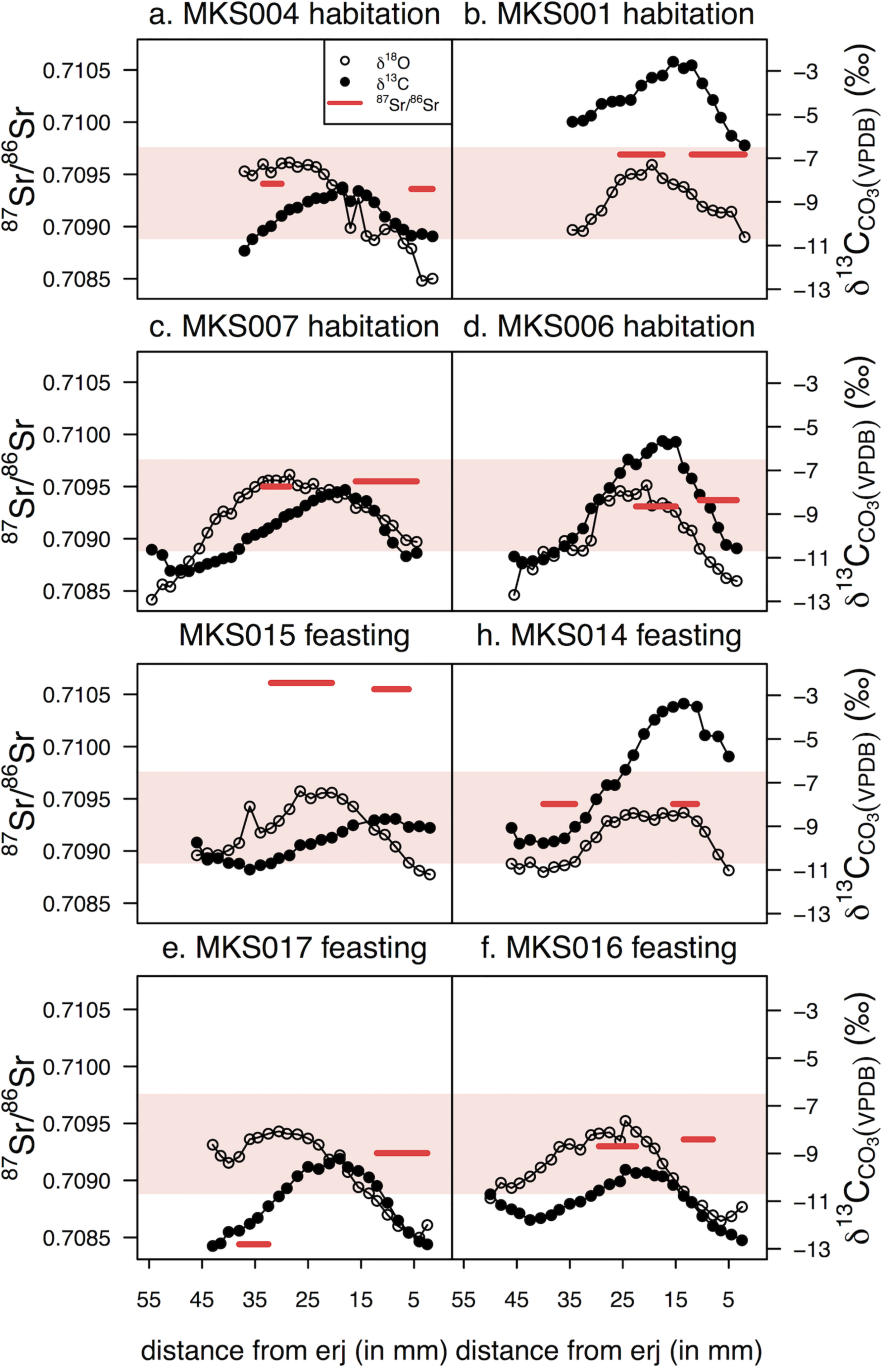
δ^18^O and δ^13^C values, and ^87^Sr/^86^Sr ratios for all cattle teeth analyzed in this study. The y-axis for δ^18^O values is not shown, but the scale is the same as in Fig 11. The shaded region indicates the local range of Sr isotope ratios determined using measurements of modern vegetation from the local environment (see Figs 12 and 13).

Most of the enamel ratios lay within the local ^87^Sr/^86^Sr Sr range defined above (0.70889–0.70978), but two individuals (MKS015 and MKS017) lay outside of it. MKS015 spent the first year of its life in a location with more radiogenic Sr geology (summer ratio = 0.71061, winter ratio = 0.71055). MKS017 spent the summer in a region dominated by a less radiogenic geological substrate (0.70844) and the winter in a region compatible with the local ratios (0.70924) (see Fig 14). As the summer portion of the tooth with the non-local ^87^Sr/^86^Sr ratio is located further away from the enamel root junction and thus mineralized first, it may be suggested that this individual was born outside of the local landscape and moved to the Pierian lowlands before its first winter. Because it died at the age of 16–28 months, this individual was likely integrated into the local herds for some time before being slaughtered for the feasts.

## Discussion

### Grazing patterns of domestic herbivores

Bone collagen δ^13^C and δ^15^N values show that cattle from Makriyalos I consumed significantly higher amounts of C_4_ plants than the other animals measured (Fig 8). This may have been a result of varying physiological adaptations or of grazing in pastures that the other species visited less frequently. The most likely source of pastures rich in C_4_ vegetation was the coastal wetlands situated a few kilometers to the north and south of the site [16,27] where C_4_ plants may have been available all year round (cf [19]).

In addition to showing higher enrichment in ^13^C, the δ^13^C values of the cattle are also more variable, which indicates that some cows consumed higher amounts of C_4_ plants than others. This may have resulted from either selective management (some individuals being taken to more C_4_-rich pastures than others) and/or natural variability (the availability of C_4_ plants in different pasture areas may have fluctuated on an annual basis due to small changes in precipitation and spring/summer flooding).

Styring et al. [84] found that compound specific δ^15^N values of some of the same samples analyzed in the present study (human, *n* = 5; cattle, *n* = 5; sheep, *n* = 5; red deer, *n* = 3) showed a strong correlation between the relative amino acid profiles of domestic cattle and sheep, suggesting that the vegetation they ate was similar. However, samples for the compound specific analysis were chosen based on their proximity to the mean bulk collagen δ^15^N value of each species, and thus do not represent the individuals (specifically cattle) with a strong influence of C_4_ vegetation in their long-term diet.

It is possible that both cattle and sheep consumed some amounts of cultivated emmer or its by-product, as the mean δ^15^N value of this crop grain (1.1 ± 0.7‰) lies a trophic level below the average bone collagen δ^15^N value of these animals (cattle: 5.0 ± 1.0‰; sheep: 5.2 ± 1.0‰). The limited numbers of emmer samples measured, along with the fact that it is the only species available for analysis, preclude the interpretation of crop cultivation strategies practiced by these early farmers. Thus, it remains unclear if this crop was cultivated inside or outside of the settlement enclosure and what form it may have been made available to the animals (fallow stubble or harvested crop).

Incremental measurements of sheep and cattle tooth enamel provide an opportunity to test the possibility that the animals spent an entire year confined within the 28 ha settlement enclosure (cf [14]). In the case of sheep, this scenario is possible, as the results are compatible with these animals grazing within a narrow local environment: their maximum δ^13^C and δ^18^O values are in phase (they occur at the same position along the axis of tooth growth) and the absolute values fluctuate as expected due to annual changes in temperature and moisture (Fig 10). Two out of the four individuals consumed C_4_ plants during the summer of their first year. If C_4_ plants were available seasonally inside the settlement enclosure (possible given the presence in the archaeobotanical assemblage of the C_4_ weed *Cynodon dactylon* [22]), then the sampled sheep could have grazed within the settlement boundaries over an entire annual cycle.

The intra-tooth δ^13^C and δ^18^O sequences of the cattle are also in phase, but the sinusoidal curves they create lie further away from the line of best fit, and so they are not as ‘smooth’ as those of the sheep. This is likely the result of consumption of larger amounts of isotopically variable vegetation driven by localized fluctuations in temperature and moisture and/or varying availability of plant types in different areas of pasture. This suggests that cattle visited more diverse pasture areas than the sheep, which makes it less likely that they grazed within the settlement enclosure all year round.

In addition to exhibiting less localized grazing, the cattle were also managed in more variable ways during their first year of life compared to the sheep. Some individuals consumed C_4_ vegetation during the summer (MKS006, MKS014), one consumed C_4_ plants throughout the whole year (MKS001), and the rest subsisted on predominantly C_3_ diets when their second molars were mineralizing (Fig 11). The fact that the intra-tooth sequences of MKS001 exhibit predictable seasonal fluctuations in δ^13^C and δ^18^O values provides support for the assertion that a fresh source of C_4_ vegetation was available fairly close to the site all year round, likely in the coastal areas.

Based on the above observations, some comments can be made about the overall short- and long-term feeding habits of the two groups of domestic herbivores. The two proxies used in this study to assess dietary behavior suggest that the diets of younger and older sheep were consistent. Enamel carbonate results show that some sheep consumed C_4_ plants during the summer of their first year, and the average collagen δ^13^C values of these animals (-18.8‰) suggest that they enjoyed a small, likely seasonal, contribution of C_4_ vegetation over the long-term.

The same is not the case with the cattle. Bone collagen values show that these animals consumed varying amounts of C_4_ plants over the long-term, while their first-year seasonal dietary patterns indicate that the majority of the individuals were predominantly C_3_-feeders. The fact that younger calves exhibit lower C_4_-input than older cattle suggests that cattle of different ages were herded in distinct parts of the landscape. Such age discrimination was practiced by mid-20^th^ century ‘traditional’ farmers in Aiginio, 10 km north of Makriyalos (Halstead, field notes), who kept milking cows and suckling calves close to home, while other cattle (pregnant cows, weaned calves, oxen) were taken to coastal marshes to graze. The results presented here complement previous discussions of animal grazing patterns at Makriyalos, which have proposed both seasonal movement of livestock away from the settlement (based on the absence in limited amounts of animal dung of wild plants that set seed in summer) [92] and local herding (based on dental indications of year-round slaughter) at least of sheep and goats, perhaps integrated with arable farming [7].

### Management of feasting and non-feasting animals

The combined long-term and short-term dietary records of the domestic herbivores suggest that the management of these animals was not pre-determined by their eventual context of consumption. There are no significant differences in δ^13^C values of cattle slaughtered for the major feasts and consumed on more mundane occasions. The seasonal dietary patterns of all cattle analyzed (*n* = 8) were variable overall, but the choice of early-life management and the type of diet that calves consumed were not related to the type of context in which the animals were ultimately discarded. Similarly, neither the bone collagen values nor the seasonal intra-tooth sequences of sheep buried in feasting and non-feasting contexts show systematic differences. Thus, it is inferred that the organization of feasts did not prescribe long-term differential treatment of the domestic herbivores.

These findings do not echo the comparisons of dental microwear patterns of ovicaprids from feasting and habitation deposits [13], which suggested that animals from the two groups had somewhat contrasting diets, with feasting sheep and goats exhibiting consumption of less abrasive and more ‘fattening’ diets. However, it must be borne in mind that the temporal resolution of dental microwear (last few weeks of the individuals’ lives) is much shorter than the time represented by stable isotope values (first year or last few years of the individuals’ lives), and thus the two lines of evidence are not directly comparable.

### Long-distance movement of cattle

Two cattle individuals (MKS015, MKS017) buried in the feasting deposit show signs of non-local birth based on their strontium isotope ratios (Fig 14). This suggests that they spent a portion of their first year grazing beyond the Pieria lowland landscape.

Biologically available strontium on the Greek mainland has not yet been sufficiently mapped. Nafplioti [93] attempted to provide a first map of this type for the Aegean, but the bulk of measurements were made on archaeological tooth enamel (and a few samples of archaeological bone), which cannot be assumed to be local to the depositional environment. A small number of modern shells were also measured at some of the sites [93,94] but the results were not correlated to the underlying geological formations, rather to ‘isopic zones’, which are made up of geologies of varying ages.

The most radiogenic ratios measured by Nafplioti [93] come from archaeological sheep/goat enamel from Tharrounia (Evvia) (ratio of 0.71110) and archaeological pig and sheep/goat enamel from Agio Galas on Chios (ratios of 0.71187, 0.71053, 0.71108). Even though the early-life location of these animals is unknown, both geographical regions lie close to Paleozoic formations (metamorphosed flysch on Chios and schist on Evvia), which formed at the same time as the Upper Paleozoic–Middle Triassic schists in the Pieria Mountains and which should thus have similar ^87^Sr/^86^Sr ratios.

MKS017 was born in a region with less radiogenic ^87^Sr/^86^Sr ratios (0.70844) compared to the local landscape. Pure Cretaceous limestone is estimated, using dated marine sediments [95], to have ratios of 0.7071–0.7078, and it is possible that this individual spent the latter portion of its first year in the Mesozoic (Cretaceous, Jurassic, Triassic) limestone zones of the Pieria and Olympus mountains. Movement from these zones to the coastal lowlands would not substantially dampen the amplitude of variation in δ^18^O values of the tooth (cf [95,96]), as the limestone regions are located at elevations of only c.300 m.

MKS015 spent both the summer and the winter of its first year of life in a location with more radiogenic Sr geology (ratios of 0.71061 and 0.71055) for which the Paleozoic bedrock southwest of Makriyalos is one (but by no means the only) plausible candidate. MKS015 was killed at the age of 16–28 months, so its relocation to the Neogene lowlands happened sometime during its second or early third year of life. The Paleozoic geology is located at elevations of over 1500 m, so movement from this region would be expected to dampen the amplitude of intra-tooth variation in δ^18^O values (cf [95,96]). However, Makarewicz [96] argues–based on measurements of sequential δ^18^O values of obligate and non-obligate drinkers from southern Jordan–that simply using the amplitude of variation in δ^18^O values for assessing vertical movement leads to inconclusive results.

The Sr isotope data presented here provide evidence that two of the cows consumed during the communal feasts were not born in the local landscape and so were probably brought to the site by exchange or as a contribution from incoming participants. Grinding stones made of schist and gneiss [14] may reflect connections to the upland areas with Paleozoic schists (located c.34 km southwest of the site), while exotic obsidian in the chipped stone assemblage suggests that people at Makriyalos were part of networks reaching more distant regions of southern Greece [5]. Such material exchanges are likely to have been entangled with intermarriage and other forms of alliance between communities, for which the communal feasts may have served as an important arena.

### Contribution of cattle products to human diets

Previous bulk stable isotope analysis of the assemblage from Makriyalos I focused exclusively on the remains of humans [12]. The results showed that the people consumed distinctly terrestrial C_3_ diets, as no significant inputs of C_4_ plants or marine foods were detected in their bone collagen values. Pappa et al. (p.84) [5] argued that these C_3_ diets were based on plant protein with “a generally modest animal protein intake”, but this argument was made in the absence of measurements of crop and domestic herbivore δ^13^C and δ^15^N values.

The bone collagen results presented here show that cattle products (whether meat or milk or both) did not provide a significant contribution to the human dietary protein intake. The human isotopic values are not only predominantly terrestrial, but also strikingly non-variable in terms of their δ^13^C values. Since butchery marks and bone fragmentation data suggest that the cattle were eaten [4] and that their carcasses were processed more intensively than those of sheep, goats and pigs [97], it would seem that the animals were consumed on episodic occasions, which might not register in long-term bone collagen signatures. These occasions may have included both smaller feasts (which were cleared into everyday habitation disposal pits) as well as large-scale feasts (the remains of which were discarded in features like Pit 212).

The tooth enamel carbonate results obtained in this study were used to assess the timing of births of the domestic animals following the method established by Balasse et al. [68,98] (Vaiglova in prep). Births that are staggered throughout the year may indicate that farmers manipulate the timing of the animals’ reproductive cycle in order to ensure the presence of lactating cows and thus readily available milk during every season. At Makriyalos, the results showed that the cattle (7 out of the 8 individuals could be modeled) as well as the sheep (3 out of 4 individuals could be modeled) were all born during a narrow period of three months, suggesting that the farmers did not make effort to ensure year-round supplies of milk by extending the animals’ birthing season.

The potential role of milk use at Makriyalos was previously assessed using organic residue analysis of potsherds (*n* = 103). The results of the latter analysis provided no traces of milk residue among the extracted lipids (about 35% of the sherds analyzed contained > 5 μg g^-1^ of lipid; mean lipid concentration was 90 μg g^-1^) [99], suggesting that the sampled vessels were not used to heat milk products, although this does not preclude consumption of milk without heating in ceramic containers. The isotopic results presented here, however, offer no hint that domestic ruminants were managed to maintain a continuous supply of fresh milk, consistent with previous suggestions that animal management was generally more meat-oriented (rather than milk-oriented) in northern Greece than in the rest of the northern Mediterranean region [8,100].

## Conclusions

The isotopic work carried out in this study provides an unparalleled opportunity to examine the interplay of domestic animal herding practices, the use of animal products for human consumption and the role of long-distance exchange networks in supplementing agricultural products at an early farming site in northern Greece. Moreover, we demonstrate the successful application of a multi-isotope approach in addressing questions of animal management, which can be applied to other regions and time-periods.

On an intra-annual scale, incremental tooth enamel carbonate δ^13^C and δ^18^O values suggest that, while sheep grazed on an ecologically narrower–and thus likely more local–range of pastures during their first year of life, cattle exploited an ecologically more diverse–and perhaps spatially more extensive–catchment. Both groups of animals had access to C_4_ vegetation throughout their lives, but cattle were more likely preferentially herded in C_4_-rich pastures later in their lives, possibly in areas located closer to the coast. The management of the domestic animals was not pre-determined by their eventual context of consumption (i.e. in large feasts or smaller consumption events).

Cattle did not provide a significant long-term input to the dietary protein of humans buried in Makriyalos I, and were likely consumed on episodic small- and large-scale events. Furthermore, lack of evidence that the timing of cattle and sheep births were staggered suggests that farmers did not make an effort to secure supplies of milk throughout the whole year.

Sr isotope analysis provides evidence that two cattle individuals discarded in the Pit 212 feasting deposit were born outside the local Pierian landscape and were brought and integrated into the local herds before being slaughtered in the feasts. These non-local cattle may have been acquired through exchange networks that also brought the exotic materials used for chipped and perhaps ground stone tools at Makriyalos [5]. Maintenance of such networks provides one possible rationale for organizing these community-wide feasts.

## Supporting information

S1 File Supplementary materials and methods. Details of instrument measurement and data normalization.
(DOCX)

S1 Table Contextual information of bone collagen and plant samples measured in Stage 1. (XLSX)

S2 Table All plant and bone collagen δ^13^C and δ^15^N values obtained in Stage 1. (XLSX)

S3 Table Matching mandibular collagen δ^13^C and δ^15^N values and average intra-tooth enamel δ^13^C values of individuals analyzed in Stage 2. Standard deviation (SD) of collagen values indicates the instrument error attached to each measurement, while the SD of average enamel values indicates intra-tooth variability.
(XLSX)

S4 Table Cattle δ^13^C and δ^18^O values obtained in Stage 2. (XLSX)

S5 Table Sheep δ^13^C and δ^18^O values obtained in Stage 2. (XLSX)

S6 Table ^87^Sr/^86^Sr ratios of modern vegetation from coastal northern Pieria. The samples were collected from seven geological zones within 15 km of the archaeological site. The measurements are used to establish the ‘local range’ of ^87^Sr/^86^Sr ratios. For descriptions of the zones and location of sampling points, see Fig 6. 2σ uncertainty of the ^87^Sr/^86^Sr ratios is 0.00004.
(XLS)

S1 Fig FTIR spectrum of MKS015. (TIF)

S2 Fig FTIR spectrum of MKS104. (TIF)
