# Supplementary material for: Of cattle and feasts: Multi-isotope investigation of animal husbandry and communal feasting at Neolithic Makriyalos, northern Greece
Source: PLoS One. 2018 Jun 7;13(6):e0194474. doi: 10.1371/journal.pone.0194474 (PMC5991682; doi:10.1371/journal.pone.0194474)
Supplement: S1 File — Details of instrument measurement and data normalization. (DOCX) [file pone.0194474.s001.docx]

S1 File. Supplementary Materials and Methods

Stage 1: Collagen and charred plant analysis

δ^13^C and δ^15^N measurements of collagen were carried out in duplicate: the first set (analyzed by Rebecca Fraser, RF) was obtained at the NERC Isotope Geoscience Laboratory (NIGL) in Keyworth, UK, on a VG TripleTrap and Optima dual–inlet mass spectrometer; the second set of measurements (analyzed by Petra Vaiglova, PV) was carried out at the Research Laboratory for Archaeology and the History of Art (RLAHA), University of Oxford, on a SerCon 20/22 continuous flow mass spectrometer coupled to a Callisto elemental analyzer.

δ^13^C and δ^15^N values of plants were measured separately on the same instrument as the collagen samples at RLAHA (PV).

Measurement uncertainty was assessed based on the within-run variability of an internal alanine standard and two international reference materials for each element (IAEA–CH6 and IAEA–CH7 for δ^13^C and IAEA–N2 and USGS–40 for δ^15^N) and calculated using a two-point calibration method in R (version 3.2.2) with a script written by Erika Nitsch. The average measurement uncertainty (1σ) for δ^13^C was 0.33‰ (with a range between 0.08 and 0.93‰). The average measurement uncertainty (1σ) for δ^15^N was 0.38‰ (with a range between 0.09 and 1.10‰).

Stage 2: Enamel carbonate δ^13^C and δ^18^O analysis

Measurement of carbonate δ^13^C and δ^18^O values was carried out at the Stable Light Isotope Facility, School of Archaeological Sciences, University of Bradford, on an automated Thermo Gas Bench II device coupled to a Thermo Delta V Advantage mass spectrometer.

Measurement uncertainty was assessed using replicate measurements of two internal standards (BES, bioapatite enamel standard, and Merck CaCO_3_), and three international standards (IAEA-CO-1, IAEA-CO-8 and NBS-19). The average measurement error for all standards (1σ) was 0.15‰ for δ^13^C (from 0.01 to 0.55‰) and 0.29‰ for δ^18^O (from 0.03 to 1.11‰).

Stage 3: Modern plant and archaeological enamel ^87^Sr/^86^Sr measurement

Plant samples were first washed three times with de-ionized water to remove exterior contamination. About 200mg of each sample was weighed out into microwave containers, to which was added 2mL of 8M HNO_3_, and was then placed on a hotplate overnight to allow for the initial reaction and gas release to take place. Subsequently, 4mL of HNO_3_ and 10μL of H_2_O_2_ were added to each sample and left for 1 hour. The same step was repeated for a few minutes to check that no further reaction took place. The samples were then microwaved at 150°C for 15-20min and allowed to cool for 1-2 hours. Finally, 10μL of H_2_O_2_ was added and the containers placed on a hotplate overnight. The samples were then transferred to a calibrated cation exchange column (Dowex AG 50W–X12) to enable the separation of Sr.

The enamel powder was initially washed with acetone and then rinsed in a de-ionized water ultrasonic bath before being placed on a hotplate (also in de-ionized water) at 50°C for one hour. An ^84^Sr–enriched spike was added to the samples, which were then dissolved in 2mL of 8M HNO_3_ and converted to chloride. The samples were then transferred to a calibrated cation exchange column (Dowex AG 50W–X12) to enable the separation of Sr.

Isotope measurements were performed on a ThermoFinnigan Triton multi- collector, magnetic sector, thermal Ionization mass spectrometer at the NERC Isotope Geosciences Laboratory (NIGL) at the British Geological Survey (BGS) in Keyworth. The international standard for ^87^Sr/^86^Sr, NBS987, gave a value of 0.710251 ± 0.000005 (n = 19, 2σ) during the analysis of these samples. Uncertainty of the overall process from plant samples (including microwave dissolution) was assessed using the measurement of an apple leaf standard, NBS1486, which provided a value of 0.71393 ± 0.00004 (n = 18, 2σ).
