# Supplementary figures and images for: Of cattle and feasts: Multi-isotope investigation of animal husbandry and communal feasting at Neolithic Makriyalos, northern Greece
Source: PLoS One. 2018 Jun 7;13(6):e0194474. doi: 10.1371/journal.pone.0194474 (PMC5991682; doi:10.1371/journal.pone.0194474)

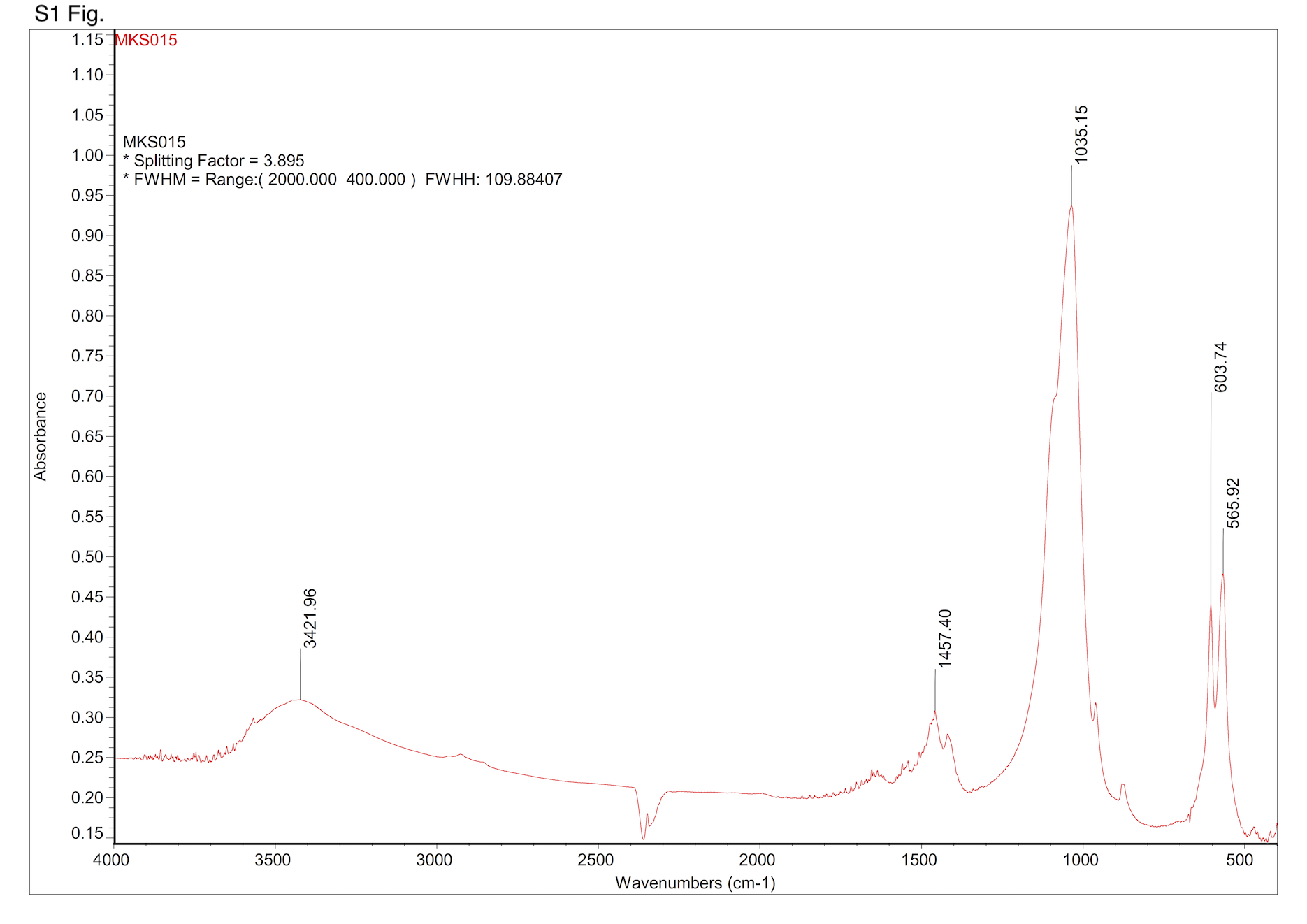

Supplement: S1 Fig — (TIF) [file pone.0194474.s008.tif]

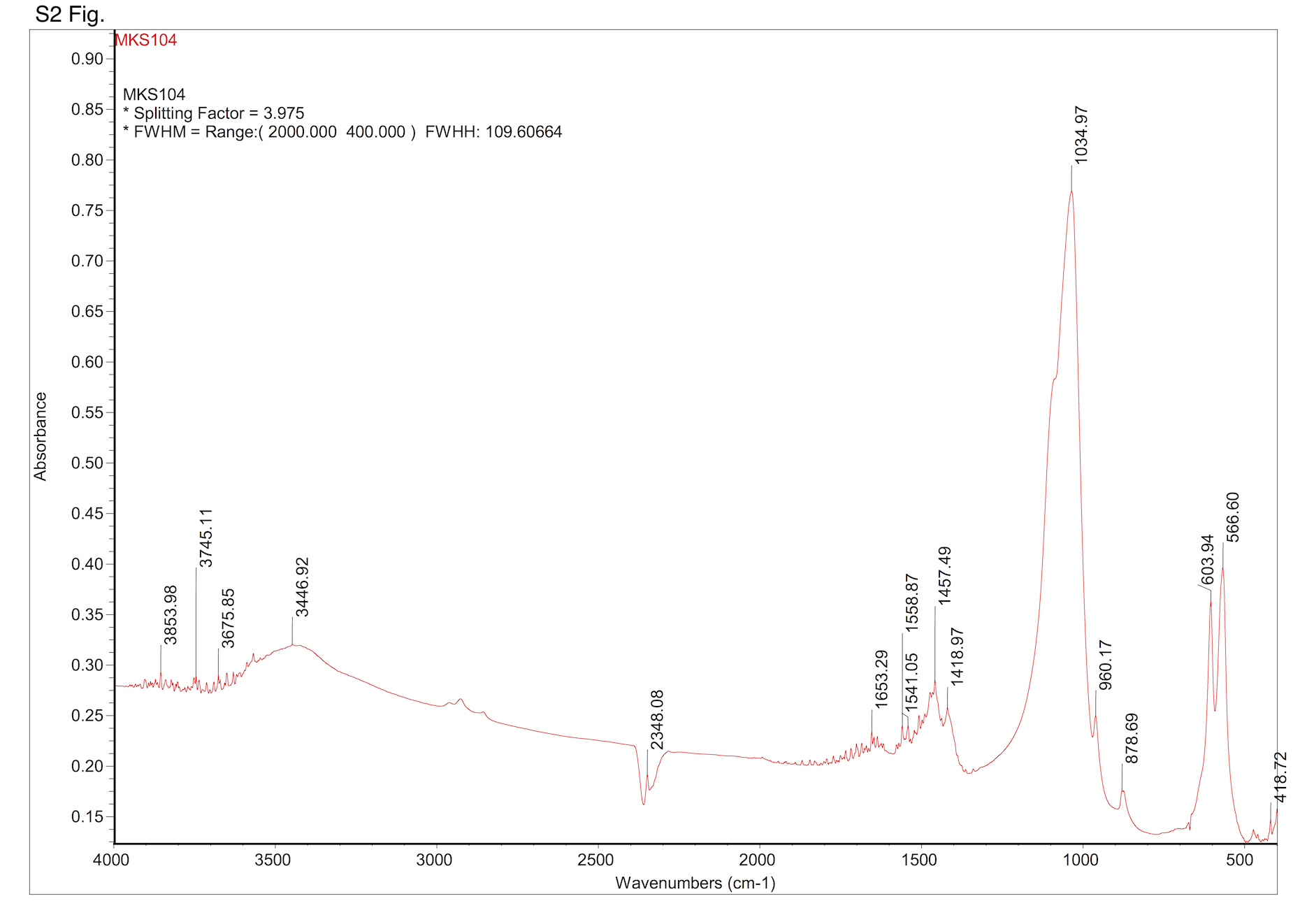

Supplement: S2 Fig — (TIF) [file pone.0194474.s009.tif]
